# M2 Macrophage‐Derived Extracellular Vesicles Reprogram Immature Neutrophils into Anxa1^hi^ Neutrophils to Enhance Inflamed Bone Regeneration

**DOI:** 10.1002/advs.202416159

**Published:** 2025-04-25

**Authors:** Yufei Yao, Yijia Yin, Fangyuan Shuai, Waishan Lam, Tao Zhou, Yaxin Xie, Xuesong He, Xianglong Han

**Affiliations:** ^1^ State Key Laboratory of Oral Diseases National Center for Stomatology National Clinical Research Center for Oral Diseases Department of Orthodontics West China Hospital of Stomatology Sichuan University Chengdu Sichuan 610041 China; ^2^ The ADA Forsyth Institute 100 Chestnut Street Somerville MA 02143 USA; ^3^ Department of Oral Medicine Infection and Immunity Harvard School of Dental Medicine Boston MA 02115 USA

**Keywords:** bone regeneration, macrophage‐neutrophil crosstalk, neutrophil heterogeneity, pro‐reparative neutrophils, single‐cell RNA sequencing

## Abstract

Periodontitis is a microbiome‐related inflammation that can lead to irreversible bone reduction and even tooth loss. This study reveals that macrophage polarization states significantly influence periodontal homeostasis, with M2 macrophage‐derived extracellular vesicles (M2‐EVs) playing a pivotal role in mitigating periodontitis‐induced bone loss. Single‐cell RNA sequencing of periodontal tissues treated with M2‐EVs uncovered a unique Anxa1^hi^ neutrophil subpopulation exhibiting pro‐reparative properties. This subpopulation is characterized by immaturity and demonstrated osteogenic and angiogenic capabilities in vivo, partially mediated through the secretion of oncostatin M (OSM) signals. The findings suggest that this functional heterogeneity arises from M2‐EVs disrupting the neutrophil maturation trajectory, with pivotal reprogramming genes, such as *Acvrl1* and *Fpr2*, driving the differentiation of the Anxa1^hi^ reparative subpopulation. This work underscores the potential of targeting M2 macrophage‐neutrophil interactions to promote the regeneration of inflamed bone tissues.

## Graphical abstract

This study reveals that M2‐EVs mitigate periodontitis‐induced bone resorption by modulating neutrophil fate. Single‐cell RNA sequencing identified an Anxa1^hi^ neutrophil subpopulation with pro‐reparative properties. M2‐EVs disrupt neutrophil maturation, promoting this subpopulation through key reprogramming genes (*Acvrl1*, *Fpr2*). These findings highlight the therapeutic potential of targeting M2 macrophage‐neutrophil interactions for bone regeneration.

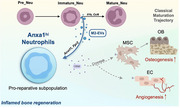

## 1 Introduction

Periodontitis is one of the most common infectious diseases in humans, impacting an estimated 20% to 50% of the global population^[^ ^1^, ^2^ ^]^ This ailment leads to microbiome‐related inflammation and progressive destruction of tooth‐supporting periodontal tissues, including the periodontal ligament and alveolar bone, which may further result in tooth mobility, eventual loss and reduced quality of life.^[^ ^3^ ^]^ Beyond being a localized oral health issue, periodontitis is also associated with several chronic disorders including diabetes, rheumatoid arthritis, and Alzheimer's disease.^[^ ^4^, ^5^, ^6^, ^7^ ^]^ Current clinical treatment strategies for periodontitis are based on the utilization of periodontal scrapers and antimicrobial agents to eliminate microbial biofilms and prevent the degradation of periodontal tissues.^[^ ^8^ ^]^ While these strategies are effective in controlling infection and inflammation, however, the difficulty in the healing of the lost periodontal structures, particularly alveolar bone, remains a critical unmet need in clinical practice.

Emerging evidence suggests that targeting the host immune response represents a promising strategy for the development of effective therapies for periodontitis.^[^ ^9^, ^10^, ^11^ ^]^ Innate immune cells, such as macrophages and neutrophils, play a critical role in maintaining periodontal homeostasis and influencing periodontal bone.^[^ ^12^, ^13^ ^]^ Macrophages are classified into pro‐inflammatory M1 macrophages, anti‐inflammatory M2 macrophages, and non‐activated M0 macrophages within local tissue microenvironments.^[^ ^14^, ^15^ ^]^ The proportion of M1 macrophages is positively correlated with the progression of periodontal, impacting the inflammatory status while promoting osteoclastogenesis and subsequent bone resorption.^[^ ^12^, ^16^ ^]^ Conversely, M2 macrophages terminate inflammatory progression through the release of various anti‐inflammatory factors, such as IL‐10 and TGF‐β, and activate osteoblasts to restore bone tissue.^[^ ^17^ ^]^ Therefore, strengthening the function of M2 macrophage may contribute to the restoration of homeostasis and inflamed bone healing.

In periodontitis, neutrophils are the first line of defense against microbial infection, they resist extracellular pathogens through phagocytosis, degranulation, and the formation of neutrophil extracellular traps.^[^ ^18^, ^19^ ^]^ While they play a protective role, exaggerated neutrophil accumulation and activation are closely linked to inflammation‐mediated bone loss.^[^ ^20^ ^]^ Recent studies have revealed that neutrophils are a heterogeneous population, capable of responding dynamically to their microenvironment.^[^ ^21^ ^]^ These cells contribute to the regulation of host immune responses and exhibit diverse functions, acting in roles that are protective, pathogenic, or involved in the resolution of inflammation.^[^ ^22^ ^]^ Neutrophils and macrophages, as the predominant leukocyte populations, are rapidly recruited to the site of infection and demonstrate a strong functional interplay during the early stages of periodontitis.^[^ ^6^ ^]^ Neutrophils exacerbate tissue inflammation by releasing proinflammatory cytokines such as IFN‐γ, IL‐1β, and IL‐6, which induce monocyte infiltration and M1 polarization.^[^ ^23^ ^]^ However, studies on their correlation in the resolution phase have been limited to the phagocytic role of M2 macrophages in clearing apoptotic neutrophils.^[^ ^24^ ^]^ The complex interactions between M2 macrophages and neutrophils during inflammation resolution and tissue regeneration remain poorly understood.

Macrophages can modulate both neighboring and distant cells by releasing extracellular vesicles.^[^ ^25^ ^]^ EVs are recognized as key regulators of intercellular communication in both physiological and pathological processes and have shown significant therapeutic potential for promoting tissue repair.^[^ ^26^, ^27^ ^]^ M2 macrophage‐derived extracellular vesicles (M2‐EVs) have been shown to modulate both the phenotype and function of recipient cells, demonstrating inhibitory effects on the generation and recruitment of M1 macrophages and pro‐inflammatory CCR2^+^ macrophages.^[^ ^28^, ^29^ ^]^ Macrophage‐derived EVs secreted after Lipopolysaccharide stimulation have been shown to promote neutrophil migration, however, the mechanisms by which M2 macrophages modulate neutrophil activity via M2‐EVs in inflamed bone regeneration remain unclear.^[^ ^30^ ^]^

Here, we found the polarization of macrophages influences the homeostasis of periodontal tissue. To enhance the effects of M2 macrophages on cellular interactions, we applied a cell‐free therapy using M2‐EVs. We performed Single‐cell RNA sequencing （scRNA‐seq) on mice periodontal bone tissue after M2‐EVs treatment and discovered dramatic changes in neutrophil proportion. We found that M2‐EVs induce the differentiation of immature neutrophils into the Anxa1^hi^ Neutrophil subpopulation and verified Anxa1^hi^ neutrophils function in tissue regeneration. Our findings demonstrated the plasticity of neutrophils after M2‐EVs administration and revealed the reparative Anxa1^hi^ neutrophil subtype in promoting inflamed bone healing.

## 2 Results

### 2.1 Macrophage Polarization State was Clinically Correlated with Periodontal Homeostasis and Disease

**Figure 1:**
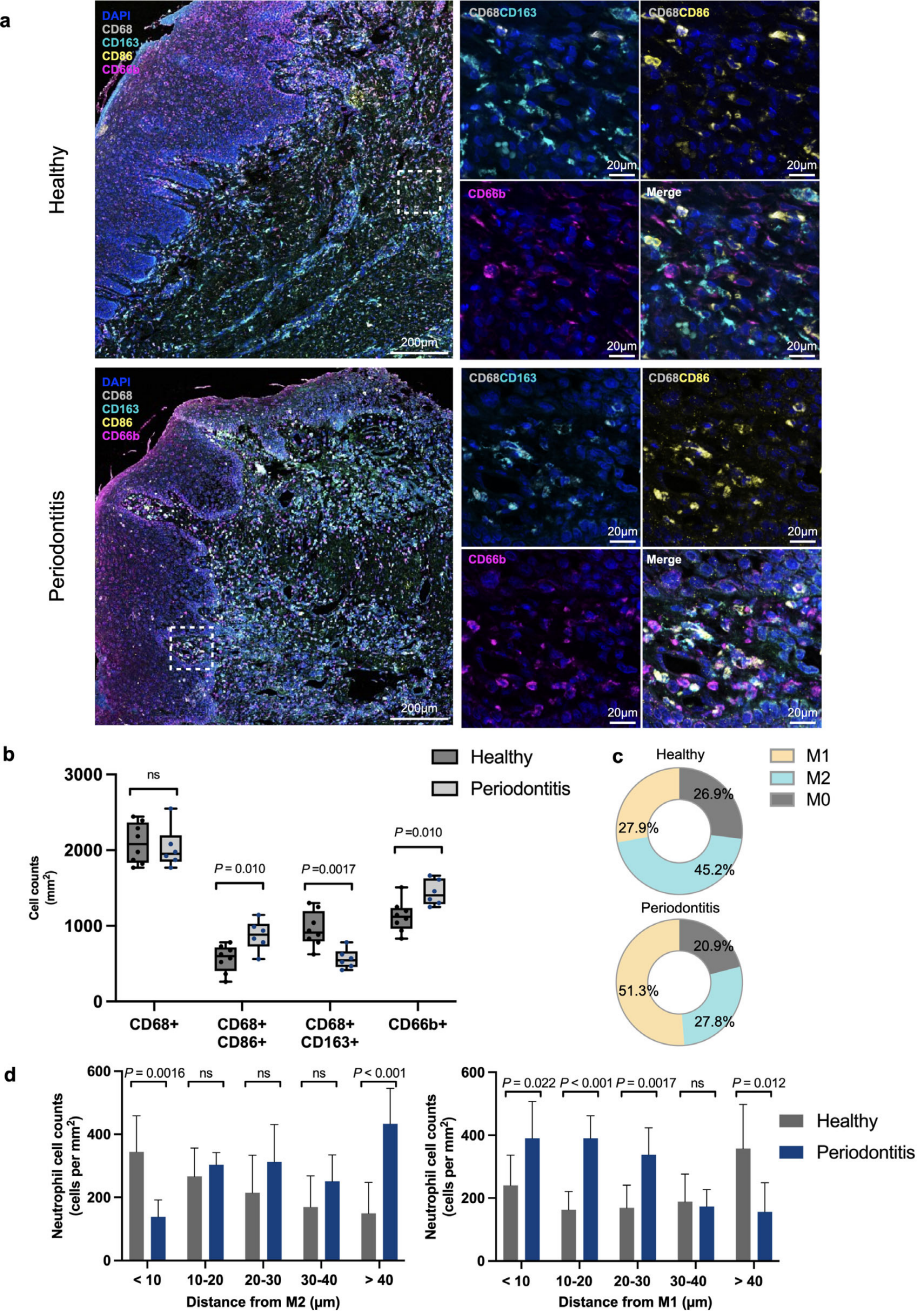
Quantification of spatial relationships of macrophages and neutrophils between healthy and inflamed human periodontal tissue. a) mIHC staining of representative periodontal tissue samples showing CD68 (gray), CD163 (cyan), CD86 (yellow), CD66b (magenta), and DAPI (blue). Scale bars, 200µm and 20 µm. b) Cell counts per mm^2^ for different cell types: macrophages (CD68^+^), M1 macrophages (CD68^+^CD86^+^), M2 macrophages (CD68^+^CD163^+^), and neutrophils (CD66b^+^). c) Pie chart revealing the proportions of macrophages in different polarization states. d) Density of neutrophils within 40 µm or above from M1 or M2 macrophage. Error bars represent means ± SD. P values as indicated by Student's t‐test. Multiplexed‐IHC analysis consisting of *n* = 8 healthy and *n* = 6 periodontitis cases.

We employed multiplex immunohistochemistry (mIHC) to investigate the spatial heterogeneity and distribution of macrophages and neutrophils in human periodontal tissue, exploring their involvement in periodontal homeostasis and disease. In comparison to healthy periodontal tissue, the periodontitis group exhibited enhanced immune cell infiltration proximal to the epithelial margins (**Figure** **1**a). While a notable increase in neutrophil (CD66b^+^) abundance was observed, there were insignificant alterations in the overall macrophage (CD68^+^) population (Figure 1a,b). Subsequently, we investigated macrophage polarization and noted a significant decrease in M2 macrophages (CD68^+^CD163^+^) and an increase in M1 macrophages (CD68^+^CD86^+^) in periodontitis samples (Figure 1b,c). This shift indicated a transition in predominant macrophage subsets from M2 to M1 macrophages from tissue homeostasis to inflammation.

We further evaluated the cell‐cell distances between macrophages and neutrophils to estimate the frequency of potential interactions. In periodontitis, the number of neutrophils within 40 µm of M1 macrophages was significantly higher, while the proximity to M2 macrophages was reduced compared to the healthy group, suggesting periodontitis enhances interactions between M1 macrophages and neutrophils while disrupting those of M2 macrophages (Figure 1d).

These findings indicate that dominant macrophage subsets are correlated with either the maintenance of homeostasis or the progression of inflammation, potentially mediated through their interactions with neutrophils. Consequently, we aim to enhance the functions of M2 macrophages and their intercellular correlation by employing M2‐EVs as a therapeutic cell‐free strategy to restore periodontal homeostasis.

### 2.2 M2‐EVs promoted inflamed bone healing in periodontitis mice model

We polarized macrophages to an anti‐inflammatory phenotype (M2) and isolated M2 and M0 (naïve control) macrophage‐derived extracellular vesicles (M2‐EVs and M0‐EVs) via ultracentrifugation (Figure S1a,b, Supporting Information). Transmission electron microscopy revealed that the isolated microvesicles had a round, cup‐shaped morphology, and Nano flow cytometry measurements showed a particle size of ≈65 nm (Figure S1c,d, Supporting Information). Bicinchoninic Acid Assay determined total protein concentrations of 0.75 ± 0.01 mg mL^−1^ (M2‐EVs) and 0.66 ± 0.07 mg mL^−1^ (M0‐EVs) (Figure S1e, Supporting Information). Western blotting confirmed high expression of EV‐specific markers CD9 and TSG101 in both M2‐EVs and M0‐EVs (Figure S1f, Supporting Information). To assess local retention, EVs were labeled with D iR Iodide and injected into the periodontal area. Fluorescence signals persisted for 48 h, indicating successful retention and activity in the local periodontal region (Figure S1g, Supporting Information).

**Figure 2:**
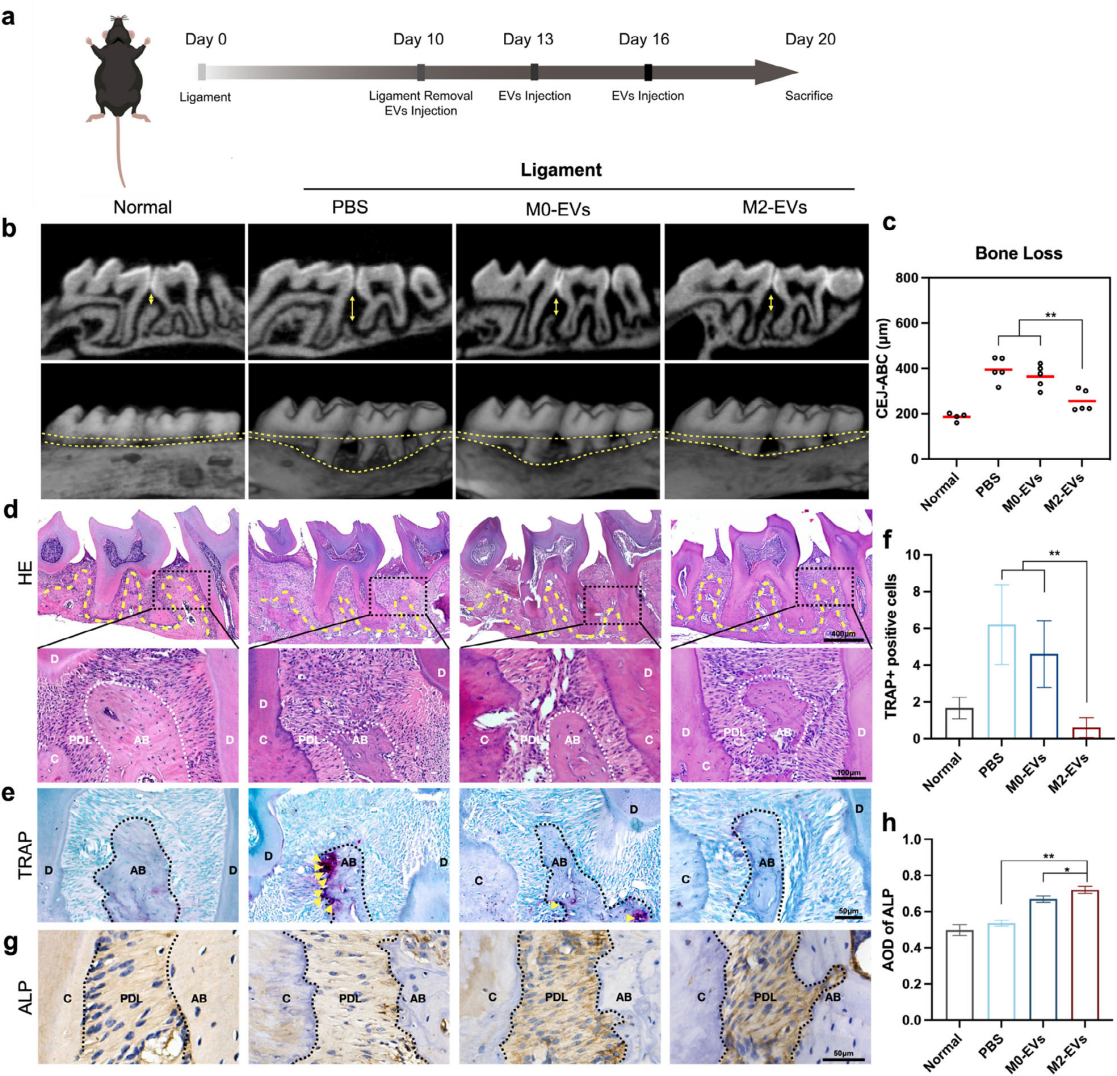
M2‐EVs promoted inflamed bone remodeling in ligature‐induced periodontitis mice. a) Schematic diagram of the in vivo study. After inducing periodontitis for 10 days, M2‐EVs, M0‐EVs, and PBS were locally administered into the gingival sulcus, with 5 µl per site at the mesial and distal gingival sulcus, for periodontitis treatment at three‐day intervals. b) Micro‐CT scanning and 3D reconstruction were performed to assess bone loss, with the yellow arrow indicating the distance between the CEJ‐ABC. c) Bone loss was assessed at the interproximal regions of the first molar (M1) and second molar (M2) of the second molar, as measured by the distance of CEJ‐ABC. d) H&E staining of periodontal tissues after treatment. Scale bar = 400 µm and 100 µm. e) TRAP staining between first and second molars. The yellow arrows indicate trap‐positive osteoblasts. Scale bar = 50 µm. f) Quantitative analysis of TRAP‐positive cells. g) Immunohistochemical staining of ALP. Scale bar = 50 µm. h) Average optical density (AOD) value analysis of ALP. PDL: periodontal ligament. AB: alveolar bone. C: cementum. D: dentine. Dashed lines were used to delineate the separation between alveolar bone, cementum, and dentine. Error bars represent means ± SD from independent replicates (*n *= 4 or 5). ns, not significant, *p < 0.05; **p < 0.01.

To evaluate the role of M2‐EVs during the post‐inflammatory phase of tissue healing, maxillary second molars of C57BL/6 mice were ligated for 10 days, and M2‐EVs were periodontally administered to the palatal gingival sulcus in second molars every three days after ligament removal (Figure **2**a). Alveolar bone loss was measured and analyzed using micro‐CT analysis, considering the distance from the enamel‐cementum junction (CEJ) to the alveolar bone crest (ABC) at the interproximal regions of the first molar (M1) and second molar (M2). Lower levels of bone loss indicated that M2‐EVs treatment efficiently mitigated attachment loss, while the PBS and M0 group showed severe bone loss (Figure 2b,c).

To elucidate histological alterations in periodontal tissue, we conducted histopathological morphological staining. As depicted in H&E staining (Figure 2d), the alveolar ridge height significantly decreased in the PBS group and M0‐EVs group, accompanied by inflammatory cell infiltration and epithelial destruction. Conversely, M2‐EVs treatment reversed these effects, leading to a restoration of alveolar bone crest height (Figure 2b,d). To further validate the osteogenic effectiveness of M2‐EVs in bone repair, we investigated the functional dynamics of osteoclasts and osteoblasts. Tartrate‐resistant acid phosphatase (TRAP) staining revealed elevated osteoclast activity in the PBS and M0‐EVs groups, which was normalized with M2‐EVs treatment (Figure 2e,f). IHC staining was employed to detect alkaline phosphatase (ALP) expression, and we confirmed an increase in the expression of osteogenic markers following M2‐EVs treatment (Figure 2g,h). Additionally, IHC analysis showed reduced expression of IL‐6 and IL‐1β in the local periodontal tissue after M2‐EVs treatment, while no significant changes were observed in serum levels (Figure S2a,b, Supporting Information).

### 2.3 Single‐Cell Expression Atlas Revealed the Increased Proportions of Neutrophils in M2‐EVs Facilitated Periodontal Bone Healing

**Figure 3:**
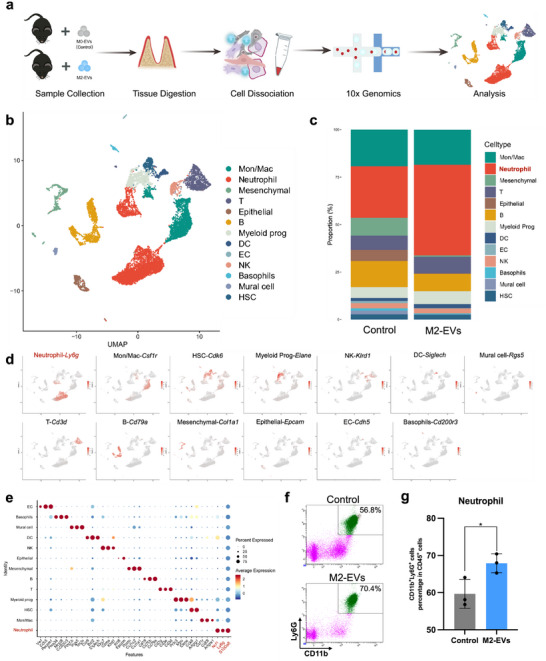
Characterization of the single‐cell atlas of periodontal tissue after M2‐EVs treatment. a) Illustration of the experimental workflow of sample preparation. b) UMAP plot of aggregated cells displaying cell clusters. *n* = 15172 cells. c) Cell ratio of different cell clusters. d) Expression profiles of selected marker genes for each cell type are mapped onto the UMAP plot. e) Dot plot of selected marker genes for each cell type. f) Original flow cytometry plots gated on CD11b^+^Ly6G^+^ to show the percentages of neutrophils in periodontal bone tissue. g) Quantitative analysis of flow cytometry to assess the proportion of neutrophils. Error bars represent means ± SD from independent replicates (*n* = 3). ns, *p < 0.05.

To explore the osteoimmunological alterations involved in periodontal tissue following M2‐EVs treatment, we harvested cells from the periodontal tissue in C57BL/6 mice following a 10‐day M2‐EVs treatment, compared to M0‐EVs treatment (serving as the control) (**Figure** **3**a). Afterquality control and filtering, we captured a total of 8316 cells in the control group and 6856 cells in the M2‐EVs group. The median gene and unique molecular identifier (UMI) counts were 1850–2078/Cells and 6362–6464/Cells, respectively (Figure S3a, Supporting Information). Unsupervised Uniform Manifold Approximation and Projection (UMAP) clustering delineated 13 distinct cell populations, identified based on established lineage markers (Figure 3b,c), including Neutrophil (*Ncf1*, *Ly6g*, and *S1008a*), Monocyte/Macrophage (*Csf1r*, *CD68*, and *Adgre1*), Mesenchymal Stem Cell (*Postn*, *Col1a1*, and *Col1a2*), T cell (*Cd3g*, *Cd3e*, and *Cd3d*), Epithelial (*Krt8* and *Epcam*), B cell (*Cd19*, *Cd79a*, and *Cd79b*), Myeloid progenitor (*Prtn3*, *Mpo*, and *Elane*), Dendritic cell (*Ccr9*, *Bst2*, and *Siglech*), Endothelial cell (*Vwf*, *Cdh5*, and *Pecam1*), NK cell (*Nkg7*, *Gzma*, and *Klrd1*), Basophil (*Prss34*, *Mcpt8*, and *Cd200r3*), Mural cell (*Pdgfrb*, *Rgs5*, and *Tagln*), and Hematopoietic Stem Cell (*Cdk6* and *Adgrg1*) (Figure 3d,e). Gene Ontology (GO) enrichment analysis revealed that M2‐EVs downregulated inflammatory response (Figure S3b, Supporting Information). However, we noted a significantly increased relative abundance of neutrophils in the M2‐EVs treatment group with a decreased expression of inflammatory‐related genes such as *Il1b*, *Cxcl2*, and *Ifitm2* (Figure 3c; Figure S3c, Supporting Information). Flow cytometry confirmed the increased amounts of neutrophils, with the proportion of CD11b^+^Ly6G^+^ cells in the M2‐EVs group increased to 67.9% ± 1.9%, compared with 59.6% ± 3.5% after M0‐EVs treatment (Figure 3f,g; Figure S3d, Supporting Information).

Excessive recruitment of neutrophils is a well‐documented factor that exacerbates inflammation and contributes to periodontitis progression.^[^ ^20^ ^]^ However, in our study, we observed an increase in the proportion of neutrophils even as the overall inflammatory levels decreased (Figure 3c; Figure S3b,c, Supporting Information). This finding suggests that neutrophils may exhibit functional heterogeneity, with certain subpopulations potentially contributing to tissue repair in addition to their pro‐inflammatory roles.

### 2.4 Anxa1^hi^ Neutrophils Exhibited Pro‐Reparative Property in Postinflammatory Periodontal Tissue

**Figure 4:**
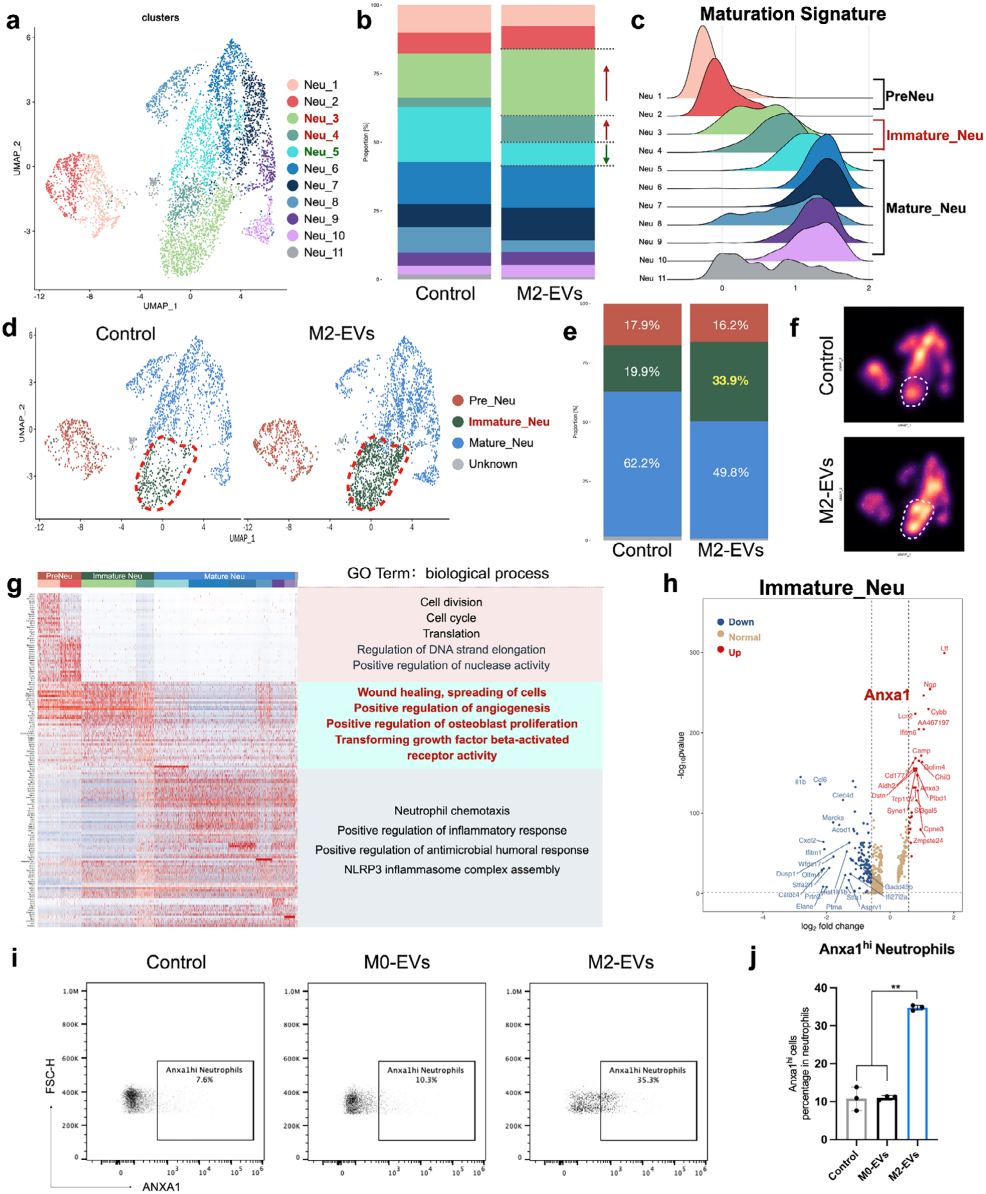
Anxa1^hi^ neutrophils exhibited pro‐regenerative gene features following M2‐EVs stimulation. a) UMAP plot of aggregate cells displaying 11 neutrophil clusters. b) Cell ratio of different cell subpopulations. c) The neutrophil maturation score of each cluster was determined based on the maturation signature.^[^ ^32^ ^]^ d) UMAP plot colored by inferred cluster identity. e) Cell ratio of Pre_Neu, Immature_Neu, and Mature_Neu in each group. f) UMAP density plot. g) Heatmap showing the row‐scaled expression of the 20 highest DEGs (Bonferroni‐corrected P values < 0.05; Student's t‐test) per cluster and GO analysis of DEGs for each cluster. h) Volcano plot displays differential gene expression in Immature_Neu compared with Pre_Neu and Mature_Neu. i) Flow cytometry plots were gated on Anxa1^hi^ neutrophils. j) Quantitative analysis was performed to assess the proportion of Anxa1^hi^ neutrophils. Error bars represent means ± SD from independent replicates (*n* = 3).**p < 0.01.

To further explore this potential dual functionality, we conducted UMAP analysis on neutrophils and identified 11 neutrophil subpopulations, labeled Neu_1 through Neu_11 (**Figure** **4**a,b). We observed an increase in the abundance of Neu_3 and Neu_4 subpopulations, while a decrease was noted in Neu_5 following M2‐EVs treatment (Figure 4b).

Integrated with a previous dataset that distinguishes neutrophils based on maturation gene signature, each cluster in our dataset was graded by maturation score from Neu_1 to Neu_11 and Figure S4a, Supporting Information).^[^ ^31^, ^32^ ^]^ Neu_1&2 exhibited maturation scores comparable to pre‐neutrophils (Pre_Neu), Neu_3&4 shared similarities with immature neutrophils (Immature_Neu), whereas Neu_5‐10 were correlated with mature neutrophils (Mature_Neu) (Figure 4c; Figure S4b, Supporting Information). The transcriptional features of Neu_11 did not align with developmental traits and was not included in further study, termed Unknown_Neu.

We further analyzed neutrophil subpopulations according to their maturation states. Immature_Neu cells showed a significant increase from 19.9% to 33.9%, and Mature_Neu experienced a decrease after M2‐EVs treatment (Figure 4d–f). Subsequent examination of the genetic and functional characteristics of each subpopulation unveiled that Mature_Neu was involved in inflammatory responses, chemotaxis, and microbiome killing, demonstrating prominent gene expression of *Nlrp3*, *Il1b*, and *Ccl6* (Figure 4g; Figure S4c, Supporting Information). In Mature_Neu, the proportion of Neu_5 (high expression of *Nos2*) underwent the most significant changes (Figure S5a, Supporting Information), and flow cytometry of periodontal tissue confirmed a decrease in the proportion of Nos2^+^ neutrophils (Figure S5b,c, Supporting Information).

Notably, except for gene expression associated with secondary granules in Immature_Neu, reparative‐related genes such as *Anxa1*, *Acvrl1*, and *Lta4* *h* were also enriched (Figure 4g,h; Table S1 (Supplementary Table S1). GO analysis unveiled pathways associated with wound healing, angiogenesis, osteoblast proliferation, and TGF‐β release in Immature_Neu (Figure 4g). To evaluate the osteogenic and anti‐inflammatory potential of immature neutrophils treated with M2‐EVs, neutrophils were isolated from bone marrow, pretreated with M2‐EVs, and then co‐cultured with bone marrow stem cells (BMSCs). This co‐culture system showed enhanced osteogenic activity, as evidenced by increasedALP activity (Figure S6a,b, Supporting Information). Additionally, M2‐EVs treatment led to a reduction in the formation of NETs (Figure S6c, Supporting Information).

Given the pro‐reparative roles exhibited by Immature_Neu, we conducted a more detailed investigation into its characteristics. Volcano analysis revealed that *Anxa1* expression had a high LogFC between Immature_Neu and other neutrophils (Figure 4h). Flow cytometry and immunofluorescent (IF) staining revealed an increased presence of Anxa1^hi^ neutrophils following M2‐EVs treatment (Figure 4i,j; Figure S7a, Supporting Information). Besides, transcription factor analysis suggested that the upregulation of transcription factors such as E2f2, Klf5, and Nfe2 may be linked to the development of the Anxa1^hi^ neutrophil subpopulation (Figure S7b, Supporting Information).

### 2.5 Immature Neutrophils were Blockaded in the Maturation Trajectory and Reprogrammed to Acquire a Pro‐Reparative Phenotype

Gene expression of immature neutrophils could be enriched in wound healing‐related pathways, which is inconsistent with the classical role of mature neutrophils as the pro‐inflammatory population. Therefore, we hypothesized that Immature_Neu does not follow the single developmental continuum, instead, it may undergo reprogramming between distinct cell states.

**Figure 5:**
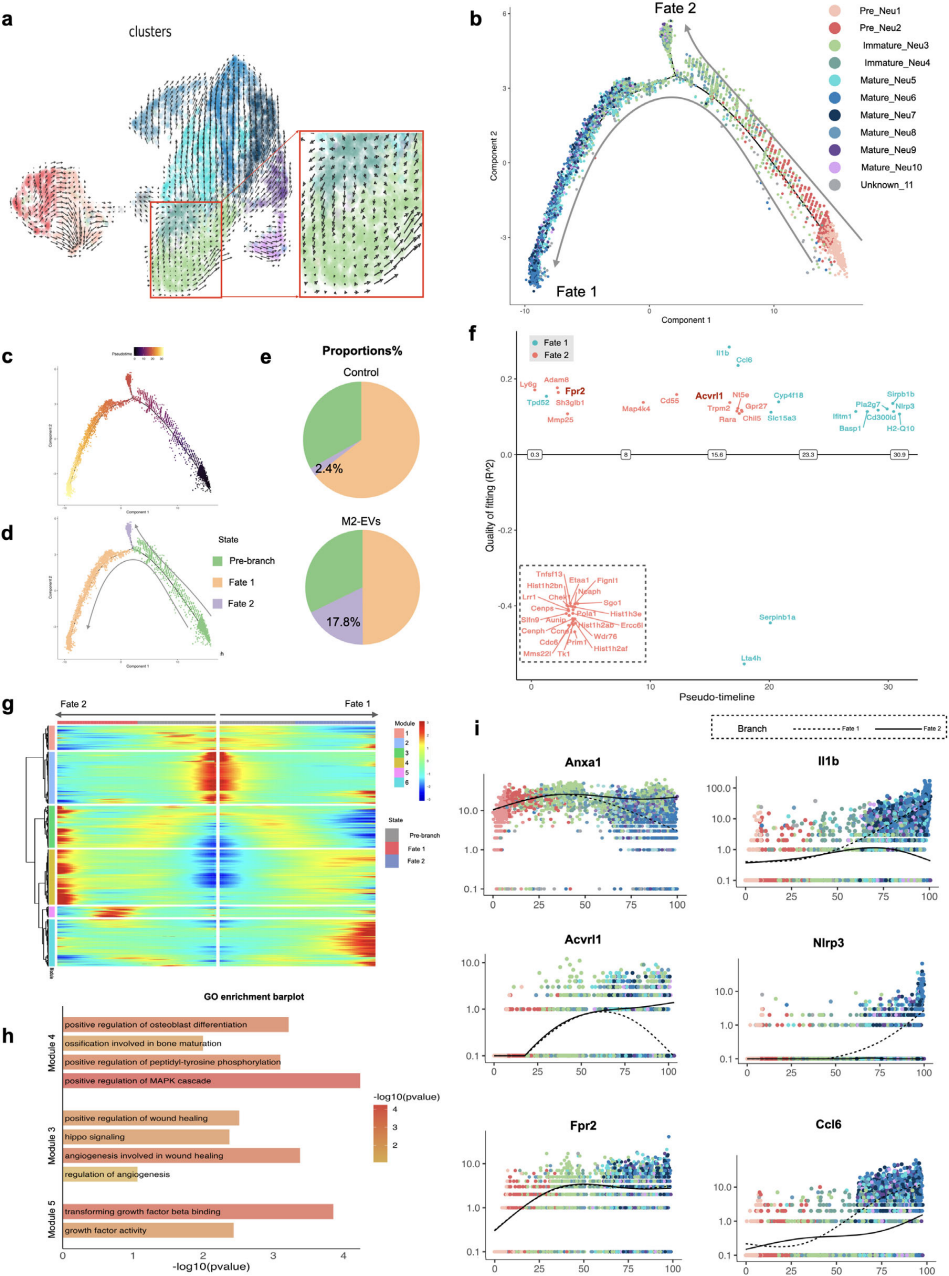
Reprogramming of Immature_Neu into pro‐reparative state. a) Projection of RNA velocity fielding onto the UMAP plot. Immature_Neu presenting inactive state (red box). b) Monocle trajectory of the neutrophil population by cluster identity. c) Cell trajectory on pseudotime value. d) Monocle trajectory colored by cellular fates. e) Pie chart revealing cell proportions of each fate. f) GeneSwitches analysis following monocle trajectory depicted upregulated gene sets above the zero line and downregulated genes below the line. The dashed box represented downregulated gene sets in fate 2. g) Pseudotime‐ordered single‐cell expression trajectories. h) Enriched GO terms of reparative state‐related modules. i) Pseudotime‐dependent genes in the differential branches. [Correction added on April 30, 2025, after first online publication: figure 5 image is updated in this version.]

To validate this hypothesis, RNA velocity analysis was performed to investigate potential cellular differentiation trajectories, Immature_Neu exhibited low RNA velocity, suggesting an inactive cell differentiation state (**Figure** **5**a). Subsequently, monocle2 was employed to arrange the neutrophil subpopulations in an unbiased pseudo‐temporal manner. While neutrophils have classically been regarded to follow a linear maturation path from pre_Neu to mature_Neu, our study revealed dual fates for neutrophils, characterized by two distinct and organized developmental trajectories (Figure 5b–d). One trajectory followed the classic maturation developmental path, ultimately differentiating into Mature_Neu, which we designated as fate 1. The other trajectory, however, is blocked in the maturation path, leading to fate 2. Comparing the proportion of neutrophils transitioning into fate 2 between both groups, it was observed that the M2‐EVs treated group demonstrated a significant increase from 2.4% to 17.8% (Figure 5e).

We then performed GeneSwitches analysis to elucidate gene sets contributing to the trajectory transition. As neutrophils differentiate into fate 1, effector genes such as *Il1b*, and *Ccl6* were “switching on”, which were previously reported to support neutrophil maturation sequencing and leading to classical pro‐inflammatory neutrophil phenotype (Figure 5f). In a similar temporal sequence, reparative genes including *Acvrl1* and *Fpr2* were “switching on”, facilitating the transcriptional reprogramming of immature neutrophils into a pro‐reparative state (Figure 5f). Concurrently, our analysis unveiled the early “switching off” of cell cycle and histon family genes along the trajectory into fate 2, resulting in the blockade along the classical maturation trajectory (Figure S8a, Supporting Information). This developmental trajectory aligned with the quiescent state transition identified by RNA velocity (Figure 5a).

We subsequently sought to distinguish the molecular changes in neutrophil fates based on pseudotime. A total of 2113 genes were measured across six modules with expression changes between the pre‐branch and two terminal fates in neutrophils (Figure 5g). We distinguished modules 3&4&5 were highly related to the dynamic expression changes along the trajectory of fate 2, as a reparative state (Figure 5g). Gene sets (*Anxa1, Anxa3, Arfgef1, Mrtfa, Flna, Stk4*, and *Reck*) in Module 3 specifically contributed to the biological process of wound healing and angiogenesis and were enriched in the hippo pathway. Genes identified in Module 4, such as *Bmpr1a, Runx2*, and *Mapk3*, contributed to the bone formation process of the regenerative state. Module 5 included genes (*Postn, Ltbp4*, and *Bmp2*) that were highly related to anti‐inflammatory factors release (Figure 5h; Figure S8b (Supporting Information) and Table S2 (Supplementary Table S2). We also mapped the fate‐determining genes onto the pseudo‐time‐dependent framework (Figure 5i). These findings thus corroborated earlier analyses that Immature_Neu possessed wound healing characteristics because it had been reprogrammed with reparative features.

To investigate the underlying mechanisms by which M2‐EVs influence neutrophils, in vitro co‐culture experiments were conducted. Neutrophils were isolated from bone marrow, treated with M2‐EVs, and subsequently purified using Fluorescence‐Activated Cell Sorting (FACS) to isolate highly active cells, followed by bulk RNA sequencing (Figure S9a–c, Supporting Information). Our results revealed that transcription factors such as E2f2, Klf5, and Nfe2 were upregulated following M2‐EV treatment, which was consistent with those transcriptional factors contributing to the Anxa1^hi^ neutrophil subpopulation (Figures S7b and S9d, Supporting Information). The upregulation of these transcription factors may directly contribute to the increased proportion of Anxa1^hi^ neutrophils. Additionally, KEGG pathway enrichment analysis suggested that M2‐EVs likely modulate neutrophil populations through the PI3K‐Akt pathway, a key signaling pathway involved in immune cell differentiation and function (Figure S9e, Supporting Information).

### 2.6 CellChat Analysis Revealed Crosstalk Between Neutrophils and Stromal Cells Via OSM Signaling

**Figure 6:**
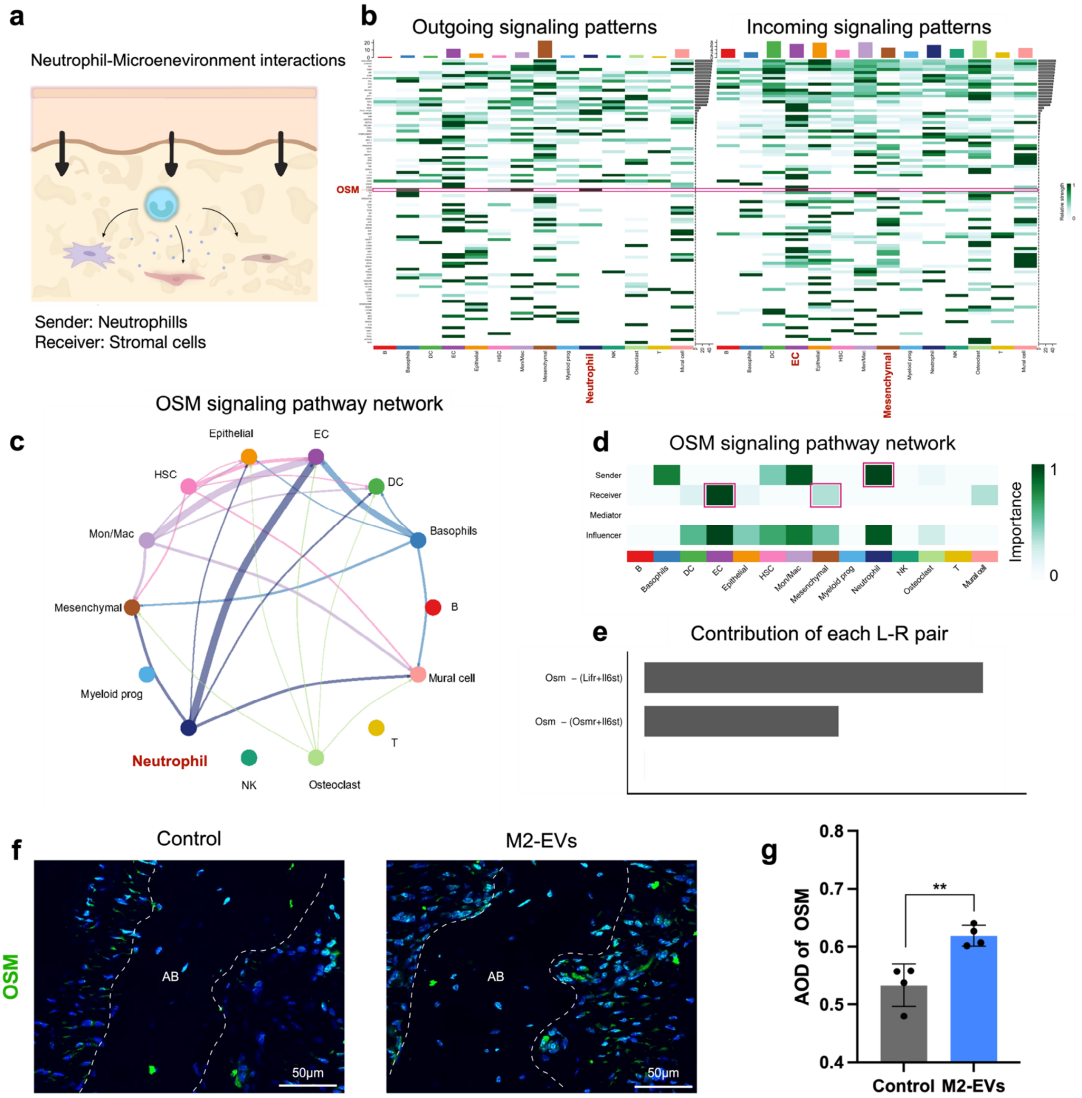
Cellular interaction between neutrophils and stromal cells mediated by OSM Signaling. a) Illustration of ligand‐receptor interactions between neutrophil and stromal cells in periodontal tissue. b) Outgoing and incoming signal patterns among all cell types. The magenta box indicated the outgoing and incoming signaling in the OSM pathway. c) OSM signaling pathway network in cell populations. d) Heatmap depicting the roles of cell types in the OSM signaling pathway. e) Bar plot illustrating the involvement of each ligand‐receptor pair in the OSM signaling pathway. f) Immunofluorescence staining of OSM, Scale bar = 50 µm. AB: alveolar bone. g) Quantitative AOD value analysis of OSM. Error bars represent means ± SD from independent replicates (*n* = 4). **p < 0.01. by Student's t‐test.

After defining the reparative‐related state of Anxa1^hi^ neutrophils, we further attempted to reveal the interaction between neutrophils and other cell types by CellChat analysis (**Figure** **6**a). Several signaling pathways that significantly influenced the outgoing and incoming signaling patterns of periodontal bone tissue were identified (Figure 6b). We found that the Oncostatin M (OSM) pathway was especially activated among neutrophil outgoing signals. Neutrophils were the main sender of the OSM signal, while Mesenchymal cells (MSCs), endothelial cells (ECs), and vascular mural cells were the recipients, which aligned with the recognized roles of neutrophils in angiogenesis, ossification, and tissue healing (Figure 6c,d). We further explored the ligand‐receptor pairs and identified the OSM‐OSMR/IL6ST and OSM‐LIFR/IL6ST complexes (Figure 6e). In comparison to the control group, we observed increased expression levels of OSM in periodontal tissue (Figure 6f). The crosstalk between neutrophils and stromal cells were mediated by OSM signaling, indicating that neutrophils influence stromal cells to promote tissue regeneration.

### 2.7 Neutrophils pretreated with M2‐EVs exhibited enhanced functions in osteogenesis and angiogenesis during periodontal bone healing

**Figure 7:**
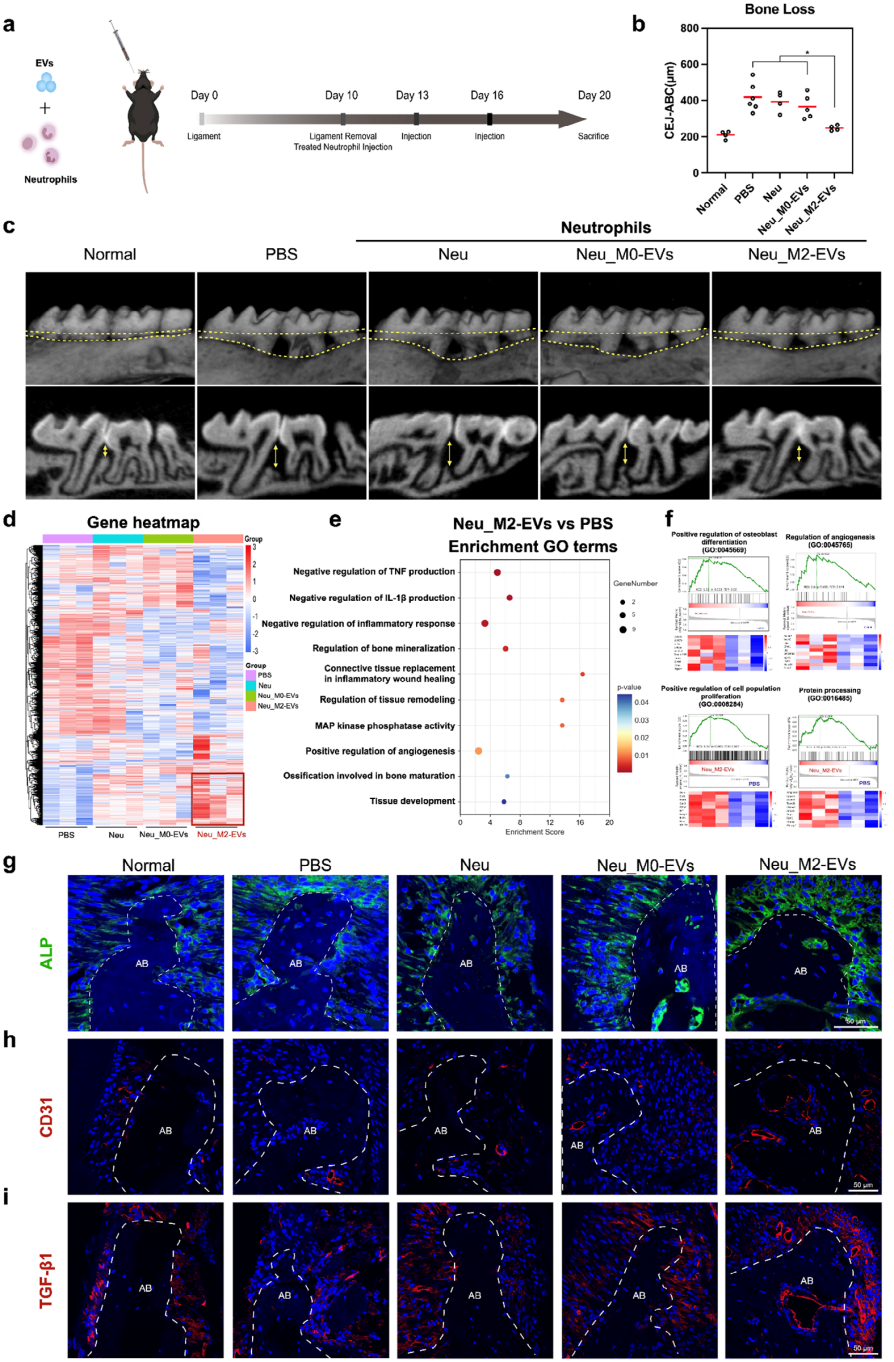
M2‐EVs stimulated neutrophils promoted bone remodeling in ligature‐induced periodontitis mice. a) Schematic diagram of the verification study. b) Bone loss was measured by the distance of CEJ‐ABC at the interproximal regions of the first molar (M1) and second molar (M2). c) Micro‐CT scanning and 3D reconstruction were performed to assess bone loss, with the yellow arrow indicating the distance between CEJ‐ABC. d) Bulk RNA‐seq analysis of differently expressed genes among PBS, Neu, Neu_M0‐EVs, and Neu_M2‐EVs group. Cutoff: p‐value < 0.05 and |log2 FC| > 2. e) GO analysis between Neu_M2‐EVs and PBS based on differentially expressed genes. f) GSEA revealed the enrichment of differently expressed genes in the regenerative‐related pathways. IF staining of ALP g), CD31 h), and TGF‐β1 i) in mouse model after treatment. Scale bar = 50 µm. Error bars represent means ± SD from independent replicates (*n* = 4–6). *p < 0.05.

To assess the impact of M2‐EV‐treated neutrophils on periodontal bone healing, neutrophils were isolated from the bone marrow of 8‐week‐old mice. Subsequently, they were pretreated in vitro with M2‐EVs for 6 h, followed by the removal of EVs through centrifugation. Following this, pretreated neutrophils were locally injected into mice with periodontitis (after ligament for 10 days) every three days as therapeutic agents (**Figure** **7**a). A significantly lower CEJ‐ABC distance in the M2‐EVs treated neutrophils (Neu_M2‐EVs) group indicated reduced bone loss (Figure 7b,c). In contrast, untreated neutrophils showed no significant effects on periodontal bone tissue. We found no systemic impact of neutrophil cell therapy on peripheral blood immune cell proportions (Figure S10a, Supporting Information). We further compared the effects of reprogrammed neutrophils with those of M2‐EVs alone. Our results demonstrated that both reprogrammed neutrophils and M2‐EVs significantly promoted alveolar bone height restoration. However, a slight advantage in bone repair was observed in the reprogrammed neutrophil group, which may be attributed to enhanced local cell‐cell interactions within the periodontal tissue (Figure S10b,c, Supporting Information).

Subsequently, bulk RNA sequencing was employed to explore the regulatory effects and underlying mechanisms of the regenerative properties. A distinct gene expression pattern was evident in the Neu_M2‐EVs group in the heatmap (Figure 7d). To further investigate the functional implications of neutrophils in periodontal bone healing, GO analysis was applied. The enriched pathways in the Neu_M2‐EVs group were highly related to the release of anti‐inflammatory factors, ossification, angiogenesis, and tissue remodeling (Figure 7e). Confirming the analysis, Gene set enrichment analysis (GSEA) revealed upregulation of the osteoblast differentiation, angiogenesis, cell population proliferation, and protein processing pathway in the Neu_M2‐EVs group compared to the PBS group (Figure 7f). We conducted qPCR validation to confirm the findings for genes of interest within the identified pathways. Specifically, we validated the expression levels of *Wnt7b, Asprv1, Efna1*, and *Hras*, which were found to be significantly upregulated in the Neu_M2‐EVs group compared to other groups (Figure S10d,e, Supporting Information).

Herein, we conducted an assessment of ossification, angiogenesis, and anti‐inflammatory factors utilizing immunofluorescence analysis. In comparison to the PBS group and the Neu_M0‐EVs group, we observed increased expression levels of ALP, CD31, CD34, VEGF, and TGF‐β1 in the Neu_M2‐EVs treated group. Additionally, the expression of ALP and TGF‐β1 was localized in the periodontal ligament, while CD31, CD34, and VEGF expression was observed both in the periodontal ligament and alveolar bone. These findings suggested an enhancement of tissue healing in the inflamed bone model (Figure 7g–i; Figure S11, Supporting Information). These results correlated with scRNA‐seq analysis, indicating the efficacy of local treatment with M2‐EVs‐treated neutrophils in mitigating periodontal bone resorption.

## 3 Discussion

Traditionally, neutrophils were regarded as a homogeneous cell population.^[^ ^20^ ^]^ However, it is now acknowledged that neutrophils exhibit considerable phenotypic and functional diversity. This heterogeneity is manifested in distinct subpopulations of neutrophils, characterized by unique phenotypic markers, transcriptional profiles, and functional properties.^[^ ^22^ ^]^ Emerging evidence suggests that neutrophils may exhibit anti‐inflammatory or regenerative characteristics in the liver, peritoneal tissue injury, and bone system.^[^ ^33^, ^34^ ^]^ Neutrophils have been categorized into pro‐inflammatory (N1) and anti‐inflammatory (N2) subpopulations, with N2 neutrophils expressing markers similar to M2 macrophages, such as *Arg1, Il10, Ym1*, and *Cd206*.^[^ ^33^, ^35^, ^36^ ^]^

In this study, we identified a distinct pro‐reparative neutrophil subpopulation with a unique transcriptional signature. This subpopulation exhibits a high expression of *Anxa1*, *Acvrl1*, and *Lta4* *h*, genes linked to anti‐inflammatory responses and tissue repair. AnxA1, in particular, is a glucocorticoid‐regulated protein crucial for resolving inflammation, acting by suppressing proinflammatory mediator release, modulating monocyte recruitment, and enhancing apoptotic cell clearance by macrophages.^[^ ^37^, ^38^ ^]^ In neutrophils, AnxA1 primarily exerts its anti‐inflammatory effects by reducing activation, promoting apoptosis, and facilitating efferocytosis.^[^ ^37^, ^39^ ^]^ These actions are largely mediated through formyl peptide receptor type 2 (FPR2), a key component of AnxA1 signaling.^[^ ^40^ ^]^ While studies suggest that AnxA1 may influence neutrophil maturation, its role appears to be controversial, with evidence indicating both its involvement in maintaining an immature state and its potential to promote differentiation.^[^ ^41^, ^42^ ^]^ Here, we found that *Anxa1* expression in neutrophils is associated with an immature phenotype. This pro‐reparative neutrophil subset displayed distinct immaturity signatures, setting it apart not only from mature, pro‐inflammatory neutrophils but also from previously characterized anti‐inflammatory subsets. While prior studies have primarily focused on functional markers, our findings highlight the link between neutrophil immaturity and their reparative potential, underscoring a previously underappreciated aspect of neutrophil heterogeneity.

Advances in single‐cell transcriptomics, coupled with RNA velocity analysis, suggest a unified developmental continum of neutrophils along the maturation trajectory.^[^ ^32^ ^]^ Yet, the existence of discrete ontogenetic stages or subpopulations of neutrophils remain unconfirmed, leaving the mechanisms underlying neutrophil heterogeneity along this trajectory poorly understood. Neutrophil maturation trajectory progresses from highly proliferative Pre_Neu to non‐proliferative Immature_Neu, culminating in functional Mature_Neu.^[^ ^32^, ^43^ ^]^ Specifically, Pre_Neu exhibits the highest proportion of cells in the S phase, while immature neutrophils abruptly arrest the cell cycle and gradually enter the G0 phase as they mature.^[^ ^43^ ^]^ This transition is accompanied by the downregulation of genes related to the cell cycle. Consistently, in our study, we found the cell cycle and related genes sets including Cell cycle^[^ ^44^ ^]^ (*Mki67, Cdk1*, *Top2a, and Ccna2*), Histon family^[^ ^45^ ^]^ (*Hist* *1h1d and Hist1h2ae*) were separately downregulated in both fate 2 (pro‐reparative) and fate 1 (pro‐inflammatory), consistent with the neutrophil maturation trajectory. Notably, these genes were downregulated earlier in fate 2, suggesting that differentiation in this subpopulation initiates sooner than in fate 1. We hypothesize that the pro‐reparative subpopulation may arise because of their ability to rapidly respond to specific microenvironmental cues (M2‐EVs stimulation). Consequently, this subpopulation likely reaches terminal differentiation earlier and ceases in the immature state.

The process by which multipotent precursors commit to lineage‐specific fates is driven by the activation of genes that define the lineage, while concurrently silencing genes that are not relevant to the chosen lineage.^[^ ^46^ ^]^ Previous studies have shown that increased expression of *Il1b* and *Ccl6* supports the neutrophil maturation sequence.^[^ ^31^ ^]^ These factors can activate neutrophils, leading to the production of inflammatory mediators and chemokines, thereby facilitating neutrophil function.^[^ ^47^ ^]^ In our pseudo‐time analysis, we found that during the differentiation process of fate 1, both *Il1b* and *Ccl6* were significantly upregulated, regulating the transition of neutrophil fate toward the pro‐inflammatory phenotype. Furthermore, in a similar temporal sequence, *Acvrl1* and *Fpr2* were upregulated in the fate 2 anti‐inflammatory neutrophil trajectory. *Acvrl1* encodes Activin A receptor‐like kinase 1 (ALK1), a member of the TGF‐β superfamily. It primarily functions in vascular endothelial cells and plays a crucial role in angiogenesis and tissue repair.^[^ ^48^ ^]^ Pro‐reparative neutrophil subpopulation may also be regulated by ALK1‐associated signaling pathways in response to the local microenvironment. *Fpr2* encodes a G protein‐coupled receptor that mediates the biological functions of various ligands, including the pro‐resolution mediator AnxA1.^[^ ^49^ ^]^ Once released, AnxA1 activates FPR2 through autocrine, paracrine signaling, driving a self‐feedback mechanism in neutrophils that regulates inflammation resolution.^[^ ^50^ ^]^ Therefore, for pro‐reparative neutrophils, genes *Acvrl1* and *Fpr2* may serve as critical mediators.

Furthermore, the downstream targets through which pro‐reparative neutrophils exert their effects have also been proposed. Neutrophils are known to engage in complex bidirectional communication with various immune cells, such as macrophages, dendritic cells, and natural killer cells, as well as stromal cells.^[^ ^33^, ^51^ ^]^ Building on this, our research revealed a specific interaction between Anxa1^hi^ neutrophils and stromal cells and clarified the direct osteogenic effects of neutrophils on BMSCs. We further revealed crosstalk between neutrophils and stromal cells mediated by the effector signal OSM. The cytokine OSM influences various biological processes, including angiogenesis and osteogenesis.^[^ ^52^ ^]^ OSM could stimulate proangiogenic factors like VEGF and angiopoietin 2 in ECs, enhancing their proliferation, migration, and capillary formation.^[^ ^53^, ^54^ ^]^ In osteogenesis, OSM enhances osteoblast activity, and inhibits the adipogenic differentiation of MSCs.^[^ ^55^ ^]^ These mechanisms enable OSM to coordinate angiogenesis and osteogenesis, ensuring vascular support and enhancing the osteoblast activity. This close coupling between vascularization and osteogenesis is essential for effective bone development and regeneration. Given the predominance of neutrophils in the bone system, our study underscores the potential benefits of focusing on reparative neutrophil subpopulations to enhance bone regeneration.

Future research should focus on refining these strategies to facilitate the clinical translation of this therapeutic approach, with an emphasis on the safety evaluation of potential therapies. Key areas of exploration could include strategies to enhance the recruitment or activity of Anxa1^hi^ neutrophils by M2‐EVs therapy, or isolating patient‐derived neutrophils for reprogramming into the Anxa1^hi^ subpopulation, followed by localized injection into periodontal tissues. Additionally, integrating Anxa1^hi^ neutrophil‐targeted strategies with existing therapies, such as biomaterial scaffolds, may further enhance therapeutic outcomes and improve clinical efficacy.

Our study has several limitations. We focus on the M2‐EVs in modulating the microenvironment and identify alterations in neutrophil subpopulations. However, the precise component of M2‐EVs responsible for the reprogramming and phenotype changes in neutrophils remained to be clarified. Previous research has identified anti‐inflammatory contents in M2‐EVs, such as miR‐155 and IL‐10 mRNA,^[^ ^56^, ^57^ ^]^ which may be responsible for the observed changes. Further investigations are required to validate the predominant factors. Additionally, the significance of OSM‐mediated neutrophil‐stromal cell interactions requires further verification.

In summary, our study delineated the phenomenon that immature neutrophils could be reprogrammed into reparative terminal states via M2‐EVs treatment. We identified significant switching genes contributing to this transition process and elucidated the functions of the induced cells in osteogenesis and angiogenesis. These insights offered a comprehensive mechanism in the heterogeneity of neutrophil subpopulations and underscored the potential therapeutic implications of mediating the interaction of M2 macrophages and neutrophils in inflamed bone pathologies.

## 4 Experimental Section

### Human Periodontal Tissue Collection

Healthy periodontal tissue samples were acquired from eight individuals, and inflamed periodontal tissue samples were obtained from six individuals diagnosed with chronic periodontitis. All participants were selected from the West China Hospital of Stomatology with their informed consent. The research protocol for this study was reviewed and approved by the Research Ethics Committee of West China Hospital of Stomatology (Permit Number: WCHSIRB‐D‐2024‐284) (Chengdu, China). All experiments involving human subjects were conducted in accordance with Helsinki Declaration.

### mIHCStaining

Human periodontal tissues were fixed, dehydrated, embedded in paraffin, and sectioned with a thickness of 5 µm. Tissue sections were baked at 72 °C for 10 min and then dewaxed and hydrated with xylene and gradient alcohol. To assess the infiltration and activation status of macrophages and neutrophils, we employed the Opal technology to perform mIHC analysis with Opal 6‐Plex Manual Detection Kit – for Whole Slide Imaging (#NEL861001KT, Akoya Biosciences). Antigen retrieval and blocking were performed, and the slides were incubated with the primary antibody for 30 min. Slides were then exposed to Opal Anti‐Ms+Rb HRP for 10 min. Before each additional antibody incubation, the steps of antigen retrieval, blocking, primary antibody incubation, and Opal Anti‐Ms+Rb HRP were carried out once again. Nuclei were stained with 4′,6‐diamidino‐2‐phenylindole (DAPI) (Solarbio, China). Images were captured using Vectra Polaris (PerkinElmer, USA). Antibodies used for immunofluorescence staining including Anti‐CD68 (#ab213363, Abcam), Anti‐CD163 (#ab182422, Abcam), Anti‐CD86 (#76755S, Cell Signaling Technology), Anti‐CD66b (#ab218740, Abcam) and Anti‐Annexin A1 (#66344‐1‐Ig, Proteintech).

### Induction of Macrophages and Phenotype Analysis

RAW264.7 macrophageswere purchased from Procell Life Science & Technology Co., Ltd. and cultured in DMEM (Gibco, USA) supplemented with 10% FBS (ExCell Bio, China) and 1% penicillin/streptomycin. RAW264.7 macrophages were stimulated with 20 ng mL^−1^ Recombinant Murine IL‐4 (#214‐14, PeproTech) for 48 hours to obtain an M2 phenotype. The CD206 expression in macrophages was analyzed by immunocytochemistry (ICC). In short, the cells were placed in a 12‐well plate, fixed with 4% paraformaldehyde, and permeabilization with 0.1% Triton X‐100. Then the cells were blocked with 5% bovine serum albumin (BSA) (Solarbio, China), followed by staining of primary antibody Anti‐CD206 (#18704‐1‐AP, Proteintech) overnight, then staining of secondary antibody Goat Anti‐Rabbit IgG H&L (FITC) (#ab6717, Abcam) and stained the nuclei with DAPI. The stained samples were imaged and analyzed under a microscope (Leica DFC 7000 T, Germany).

### Macrophage Derived Extracellular Vesicles (EVs) Isolation

Naïve (M0) macrophage and M2 macrophage‐derived EVs were isolated by ultracentrifugation. Briefly, the conditioned medium obtained from cultured macrophages was sequentially centrifuged at 500 ×g for 10 min, 2000 ×g for 20 min, and 10 000 × g for 30 min at 4 °C to remove cells, debris, and large vesicles. The resulting supernatant was filtered through a sterile 0.22 µm pore filter, followed by ultracentrifugation at 100 000 ×g for 120 min. The supernatant was carefully removed and discarded and the remaining EVs were collected for further study.

### EVs Characterization and Quantification

EVs characterization was conducted according to the guideline.^[^ ^58^ ^]^ The morphology of EVs was detected by transmission electron microscopy (TEM). EVs samples were applied on the grids followed by 2% uranyl acetate staining for 2 min. Images were captured using a Hitachi HT7700 TEM (Hitachi, Japan). The particle size distribution of EVs was analyzed by nanoparticle tracking analysis (Flow NanoAnalyzer, NanoFCM, China). Expression of the EVs markers was determined by Western blot, including Calnexin (#12 186, SAB), TSG101 (#ab125011, Abcam), and CD9 (#ET1601‐9‐50, Huabio). To detect the retention of EVs, DIR (Yeasen, China) was added to EVs suspension and injected into the periodontal region (injection coordinates are the same as below). 48 hours after injection, the retention of EVs was observed using In Vivo Imaging Systems (IVIS) (PerkinElmer, USA).

### Animal Model Establishment and Treatment

All animal experiments and the research protocol in this study were reviewed and approved by the Research Ethics Committee of West China Hospital of Stomatology (Permit Number: WCHSIRB‐D‐2023‐323) (Chengdu, China). Animal models were established successfully and then randomly divided into groups.

C57BL/6J mice (Male, 6‐week‐old) were obtained from Dashuo Experimental Animal Co. Ltd. (China) and were randomly assigned into four groups: Normal (no periodontitis), PBS (periodontitis with PBS as a blank control), M0‐EVs (periodontitis with M0‐EVs treatment as a naïve macrophage control), and M2‐EVs (periodontitis with M2‐EVs). The sample size per group ranged from 4 to 5 animals, consistent with previous studies in this field, to balance ethical considerations with statistical reliability while ensuring sufficient statistical power and minimizing variability.^[^ ^59^, ^60^, ^61^ ^]^ The mice were anesthetized with 1.25% isoflurane flow. Subsequently, a ligature (6‐0 silk) was placed around the maxillary second molar from day 0 to day 10. After 10 days, the ligature was removed, PBS, M0‐EVs or M2‐EVs (5 µL per site containing 1 × 10⁹ EV particles) was injected into the mesial and distal gingival sulcus of the second molar in experimental mice using a 33‐G needle. Injections were administered every 3 days over a period of 10 days. The mice were sacrificed and analyzed 10 days after treatment. The concentrations of the indicated cytokine in the serum were examined using ELISA kits (MM‐1011M2, mmBio, China) according to the manufacturer's instructions.

For the neutrophils‐based treatment, neutrophils were isolated from mice bone marrow and neutrophils were pretreated with M2‐EVs as described under “Neutrophil isolation and co‐culture experiments”. The cell suspension was adjusted to gingival sulcus (5 µL per site containing 1 × 10^7^ neutrophils). The experimental mice were divided into five groups: Normal (no periodontitis), PBS (periodontitis with PBS treatment), Neu (periodontitis with neutrophil treatment), Neu_M0‐EVs (periodontitis with neutrophils pretreated with M0‐EVs) and Neu_M2‐EVs (periodontitis with neutrophils pretreated with M2‐EVs). The details of the animal model treatments were as described above. A comprehensive analysis of peripheral blood parameters was conducted.

### Micro‐CT scanning and analysis

Maxillary samples were collected, fixed in 4% paraformaldehyde, and subjected to micro‐CT examination (µCT 50, SCANCO Medical AG, Switzerland) with a resolution of 10 µm at 70 kVp and 200 µA. Samples were carefully oriented during scanning to ensure consistent analysis of regions of interest (ROIs). Data processing and analysis were performed by a blinded operator to reduce potential bias. The scanned data were subsequently analyzed and reconstructed using SCANCO Medical Evaluation software (SCANCO Medical AG, Switzerland). Alveolar bone resorption was evaluated by measuring the distance between the CEJ andABC on the buccal side of the interproximal regions of the first (M1) and second (M2) molars. This measurement serves as an indicator of attachment loss as described in previous studies.^[^ ^62^, ^63^ ^]^

### Histological and Immunohistochemistry staining

The tissue samples were subsequently fixed, decalcified, dehydrated, embedded in paraffin, and sectioned with a thickness of 5 µm. The sections were baked at 65 °C for 2 h and then dewaxed and hydrated with xylene and gradient alcohol. Sections were stained with hematoxylin and eosin (H&E) (Solarbio, China), Masson's trichrome (Solarbio, China), and TRAP staining (Waco, Japan) under the manufacturer's instructions. Antigen retrieval was conducted by microwave using citric acid solution and blocked with 5% BSA. The sections were incubated overnight at 4 °C with primary antibodies Anti‐ALP (#ET1601‐21, Huabio), Anti‐IL‐6 (#AF5103, Affinity) and Anti‐IL‐1β (#bs‐0782R, Bioss). Following primary antibody incubation, the sections were stained with secondary antibodies Goat Anti‐Rabbit lgG HL (HRP) (#AB205718, Abcam) and then 3,3‐diaminobenzidine (DAB) substrate. Nuclei were stained with DAPI.

### Single‐Cell RNA Sequencing

The sequencing and bioinformatics analysis were performed by OE Biotech Co., Ltd. (Shanghai, China). Periodontal tissue was harvested from C57BL/6 mice after 10‐day treatment with M2‐EVs or M0‐EVs (3 mice per group) and molars were removed. Periodontal tissue around the incisor and behind the condyle was subsequentially cut off. Single‐cell suspension was prepared from periodontal tissue by mechanical and enzymatic dissociation. Subsequently, the obtained alveolar bone tissue was cut into small pieces (<1 mm^[3]^) and digested in 0.2% collagenase type II (Gibco, USA) at 37 °C for 60 min. The sample was then collected into a centrifuge tube through a 40‐µm filter, red blood cells were removed by lysis buffer, and the supernatant was removed after centrifugation. Subsequently, the remaining cells were resuspended in RPMI 1640 medium with 0.04% BSA. A placenta blue stain was conducted to assess cell count and viability using a hemocytometer. Cell concentration was subsequently adjusted to 700–1200 cells per µL. According to the manufacturer's instructions, the Chromium Single Cell 3′ Reagent Kit v3.1 was utilized to construct scRNA‐seq libraries. The scRNA‐seq library was generated on the Illumina NovaSeq 6000 platform.

### Preprocessing of scRNA‐seq Data

CellRanger (10X Genomics, 7.0.1) was used to map the sequencing data to the mouse reference genome (mm10), with UMI counts summarized for each barcode. The Seurat (version 4.0.0) R package was used to perform quality control on sequencing data and filter low‐quality cells. Cells were filtered out by 1) gene numbers <200, UMI <1000, and log10GenesPerUMI <0.7; 2) proportion of UMIs mapped to mitochondrial genes > 10% and hemoglobin genes > 5%). The FindVariableGenes function was employed to filter highly variable genes (HVGs), followed by Principal Component Analysis (PCA) based on the expression profile of these HVGs. The PCA results were then visualized in 2D space using UMAP. Marker gene identification was performed using the FindAllMarkers function, and the identified marker genes were visualized using the VlnPlot and FeaturePlot functions.

### RNA Velocity

The velocity Python package was used to recount the spliced reads and unspliced reads with the Python script velocity.^[^ ^64^ ^]^ Subsequently, the SeuratWrappers and velocity R package were employed to compute RNA velocity values for individual genes, with the RNA velocity vectors projected onto the UMAP embedding obtained in Seurat.

### Pseudotemporal Trajectory Analysis

The developmental pseudotime of the cluster identified as neutrophil was determined with the Monocle2 package (version 2.9.0).^[^ ^65^ ^]^ The differential GeneTest function was utilized to select ordering genes (q‐value < 0.01) that were differentially expressed across clusters and dispersed. The dimensional reduction clustering analysis was conducted with the reduce Dimension function, and the pseudotime trajectory was plotted with the orderCells function using default parameters.

### Gene switches

Following Monocle trajectory analysis, gene expression data transformed into binary states (on or off) was identified by GeneSwitches (version 0.1.0) to track the timing of gene activity changes. These pivotal genes were ranked and displayed on a pseudo‐timeline, visually representing their activation or deactivation phases.

### CellChat Analysis

R package CellChat (1.1.3)^[^ ^66^ ^]^ was used to calculate the intensity of cell‐cell interactions and communication‐based on ligand‐receptor analysis with default parameters. Significant ligand‐receptor pairs were further identified and the cell communication network was aggregated using the aggregateNet function.

### Neutrophil Isolation and Co‐Culture Experiments

Neutrophils were isolated from the femurs and tibias of 8‐week‐old male C57BL/6 mice by density gradient centrifugation according to previously published protocols.^[^ ^67^ ^]^ 1 × 10^7^ neutrophils were treated with 1 × 10⁹ EV particles M2‐EVs and M0‐EVs for 6 h. The cell suspension was centrifuged at 500 ×g for 10 min to pellet the neutrophils and remove residual EVs. Pre‐treated neutrophils were locally injected into the gingival sulcus of mice or co‐cultured with BMSCs.

BMSCs were isolated from the bone marrow of 3‐week‐old male C57BL/6 mice and were cultured in minimum essential medium alpha (a‐MEM, Gibco, USA). Stem cells were purified to P3 and placed in the lower chamber of a 12‐well plate. After stimulation, neutrophils were centrifuged and placed in the top insert of Transwell (0.4 µm, Corning) (Neutrophil: BMSCs = 1:1). After co‐culture for 12 h, the neutrophil was removed and BMSCs were cultured with osteogenic media for 7 days for further osteogenic analysis.

### Flow Cytometry

Flow cytometric analysis was performed on cells extracted from mouse periodontal tissues and neutrophils isolated from femurs. Mouse periodontal tissues were obtained as described above and digested with 0.2% type II collagenase for 60 min at 37 °C. After lysis of red blood cells, the remaining cells were dyed with zombie aqua Fixable Viability Kit (#423101, BioLegend) and blocked with Fc receptor blocking reagent (#422 301, BioLegend). The cells were incubated with monoclonal antibodies labeled with the following fluorescent dyes: CD45‐EV450 (#E‐AB‐F1136Q, Elabscience), CD11b‐FITC (#101205, BioLegend), Ly6G‐APC (#127613, BioLegend). After fixation and permeabilization, cells were incubated with intracellular marker NOS2‐PE (#696 805, BioLegend). Data analyses were carried out with FlowJo 10.0 software.

Neutrophils were isolated from the femurs and tibias of 8‐week‐old male C57BL/6 mice. After blocking with Fc receptor blocking reagent (#422301, BioLegend), the cells were incubated with Anti‐Annexin A1 (#66344‐1‐Ig, Proteintech) and Goat Anti‐Rat IgG (H+L) (Rhodamine) (#ZF‐0318, ZSGB‐BIO). Data analyses were carried out with FlowJo 10.0 software.

### Immunofluorescent Staining

The periodontal tissues were embedded in an optimal cutting temperature (O.C.T) compound and subsequently sectioned at −22 °C with a thickness of 8 µm. Antigen retrieval was performed using a quick antigen retrieval solution (Solarbio, China), and the sections were subsequently blocked with 5% BSA. The sections were then incubated overnight at 4 °C with primary antibodies, washed with PBS, and subsequently incubated for 1 hour in a blocking buffer containing secondary antibodies. Nuclei were counterstained with DAPI.

Antibodies used for immunofluorescence staining including Anti‐Annexin A1 (#66344‐1‐Ig, Proteintech), Anti‐Oncostatin M (#A6163, ABclonal), Anti‐TGF‐β1 (#21898‐1‐AP, Proteintech), Anti‐CD31 (#14‐0311‐82, Invitrogen), Anti‐ALP (#ET1601‐21, Huabio), Anti‐CD34 (#ZA‐0550, ZSGB‐BIO), Anti‐VEGF (#ET1604‐28, Huabio) Goat Anti‐Rabbit IgG H&L (FITC) (#ab6717, Abcam), and Goat Anti‐Rat IgG (H+L) (Rhodamine) (#ZF‐0318, ZSGB‐BIO)

### Bulk RNA Sequencing and Transcriptomics Analysis

Following co‐culture with M2 macrophage EVs, neutrophils were purified using fluorescence‐activated cell sorting (FACS). In brief, neutrophils were stained with the Zombie YG581 Fixable Viability Kit (#423123, BioLegend) to exclude dead cells, followed by Fc receptor blocking with a reagent (#422301, BioLegend) to minimize nonspecific binding. The cells were then incubated with fluorophore‐conjugated monoclonal antibodies against CD11b (#101205, BioLegend) and Ly6G (#127613, BioLegend) for neutrophil identification. Sorting was performed using a BD FACSAria III cell sorter, and the purity of the isolated neutrophil population was validated by post‐sort flow cytometry, consistently achieving a purity of >95%, ensuring reliability for downstream analyses.

Both the harvested neutrophils and the periodontal tissue samples treated with the reprogrammed neutrophils were collected for bulk RNA sequencing analysis. RNA sequencing was carried out using the Illumina Novaseq 6000 platform. Differential expression genes (DEGs) were evaluated using the DESeq2 Bioconductor package,^[^ ^68^ ^]^ with a gene threshold set at *p*‐value < 0.05 and foldchange > 1.5 or < 1/1.5 (for periodontal tissue, fold change > 2 or < 0.5) were differentially expressed. KEGG and GO enrichment analyses were performed on DEGs to identify significantly enriched terms in the R environment (version 3.2.0) and to generate diagrams of significant enrichment terms. To encompass all genes, GSEA was conducted using R (version 3.2.0).

### Statistical Analysis

Statistical analysis was conducted accordingly.^[^ ^69^ ^]^ Data were expressed as mean ± standard deviation (SD). Statistical analyses were performed using GraphPad Prism version 9 (GraphPad Software Inc., CA, USA). For comparisons between two groups, a two‐tailed, unpaired Student's t‐test was used. For multiple group comparisons, one‐way analysis of variance (ANOVA) followed by Tukey's multiple comparison test was applied to assess significant differences between treatment groups and the control group. Statistical significance was indicated as follows: ns (not significant), *p < 0.05, and **p < 0.01.

## Conflict of Interest

The authors declare no conflict of interest.

## Author Contributions

Y.Y., Y.Y., and X.H. performed conceptualization. Y.Y., Y.Y., F.S., T.Z., Y.X., and X.H. performed the methodology. Y.Y., Y.Y., W.L., and Y.X. performed experiments. Y.Y., F.S., W.L., T.Z., and X.H. performed data analysis. Y.Y., Y.Y., X.H., and X.H. prepare the original draft.

## Supporting information



Supporting Information

Supporting Information

Supporting Information

## Data Availability

The data that support the findings of this study are available from the corresponding author upon reasonable request.
