# Supplementary material for: M2 Macrophage‐Derived Extracellular Vesicles Reprogram Immature Neutrophils into Anxa1hi Neutrophils to Enhance Inflamed Bone Regeneration
Source: Adv Sci (Weinh). 2025 Apr 25;12(28):2416159. doi: 10.1002/advs.202416159 (PMC12302528; doi:10.1002/advs.202416159)
Supplement: Supplementary file 1 — Supporting Information [file ADVS-12-2416159-s003.docx]

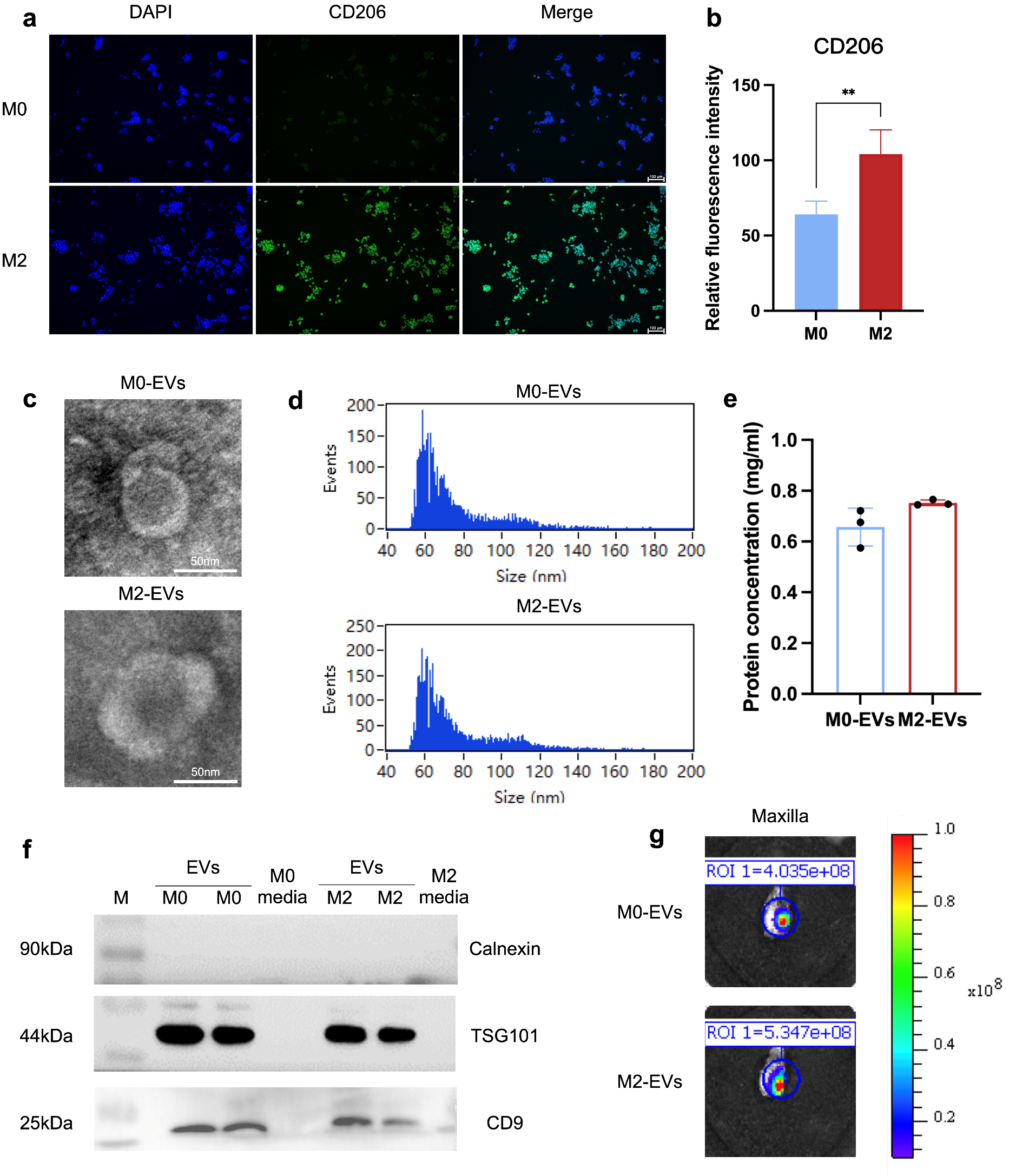


**Supplementary Figure 1. Characterization and quantification of M2 macrophage‐derived Extracellular Vesicles (M2-EVs).**

(a and b) Immunocytochemistry and relative intensity of M0 and M2 macrophages for M2 phenotype marker, CD206 (green). Scale bar = 100 µm. (c) Representative transmission electron microscope images of M0-EVs and M2-EVs morphology. Scale bar = 50nm. (d) Representative images of particle size distribution of M0-EVs and M2-EVs analyzed by nanoflow cytometry. (e) Total protein level of EVs determined by Bicinchoninic Acid assay. (f) Western blot detection to confirm presence of typical EVs markers. (g) Fluorescence image of mice maxilla 48 hours after local injection with DiR-labeled M0-EVs or DiR-labeled M2-EVs.


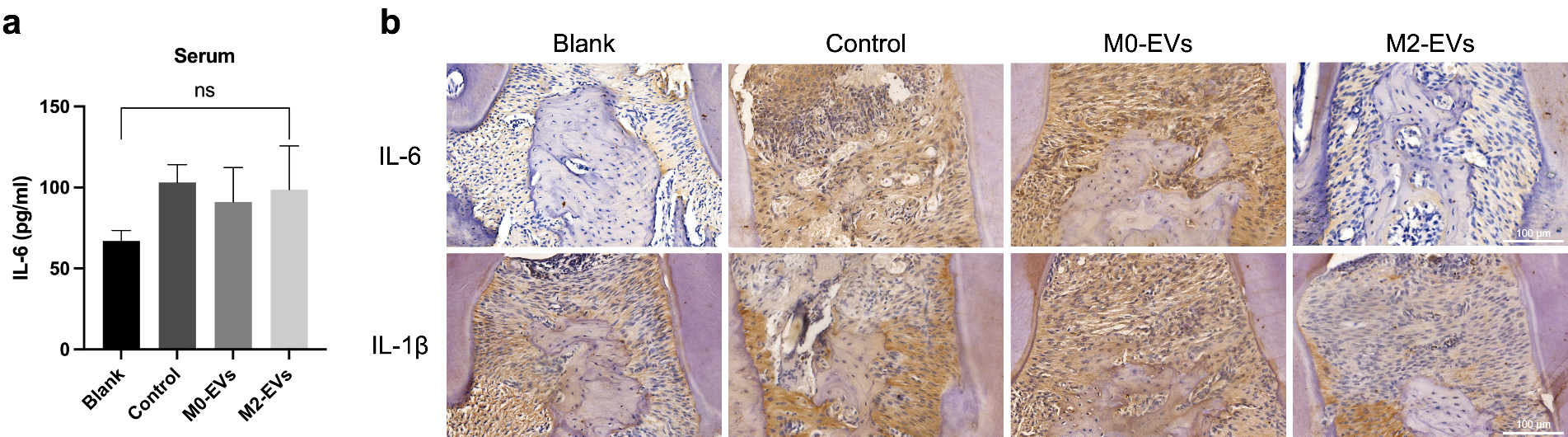


**Supplementary Figure 2. M2-EVs therapy reduces local inflammatory factor infiltration.** (a) ELISA analysis of IL-6 expression in serum. (b) Immunohistochemical staining to detect the expression of inflammatory proteins IL-6 and IL-1β in local tissue. Scale bar = 100 µm.


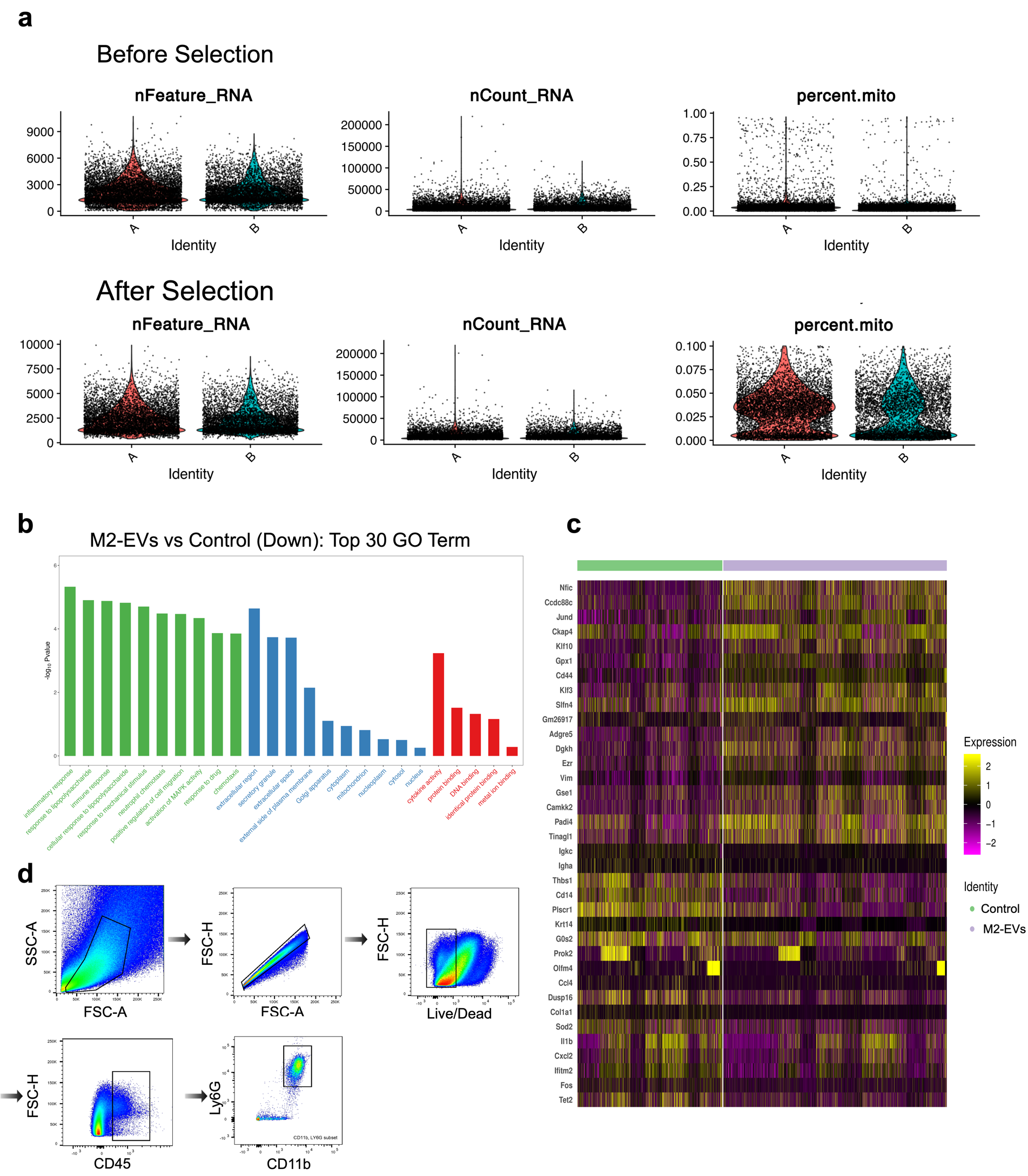


**Supplementary Figure 3.** **Quality control and comprehensive analysis of scRNA-seq data from murine periodontal tissue.** (a) Variations in sample quality were mitigated by implementing a quality control (QC) filter using Seurat. Violin plots illustrating the number of genes, the number of UMIs, and the proportion of mitochondrial genes per single cell before and after QC. (b) Downregulated Gene Ontology (GO) terms in the M2-EVs group compared to the control group. (c) Heatmap showing the expression of the highest DEGs (Bonferroni-corrected P values < 0.05; Student’s t-test) of neutrophils within each group. (d) Gating strategy for flow cytometry analysis of neutrophil populations.


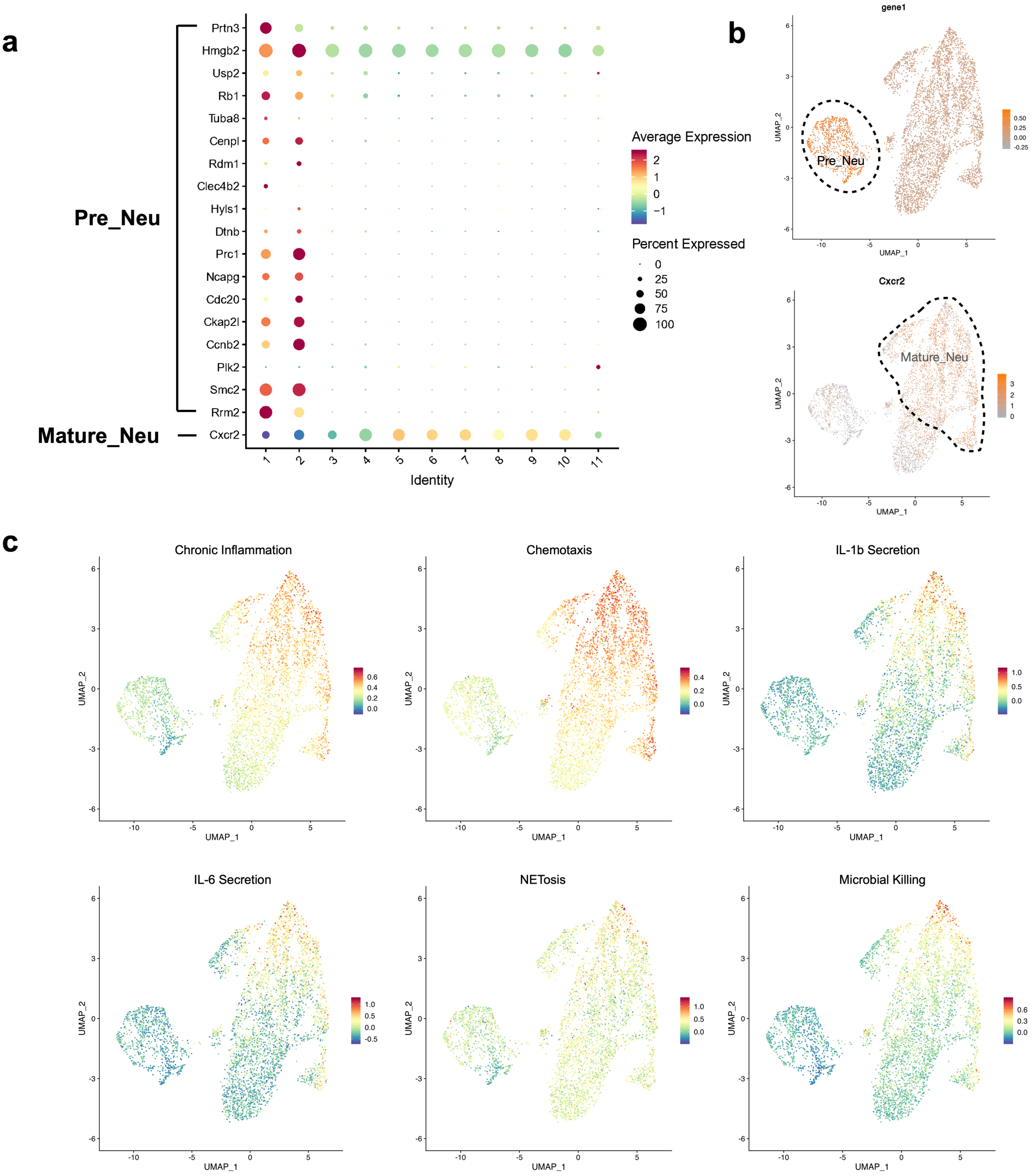


**Supplementary Figure 4.** **Identification and functional heterogeneity of neutrophil subpopulations.** (a) Dot plot classifying neutrophil subpopulations correlated with differentiation-defined gene sets. (b) UMAP plot showing maturation status in neutrophils, as scored by the AddModuleScore function. (c) UMAP plot showing representative proinflammatory characteristics in neutrophils, as scored by the AddModuleScore function.

**
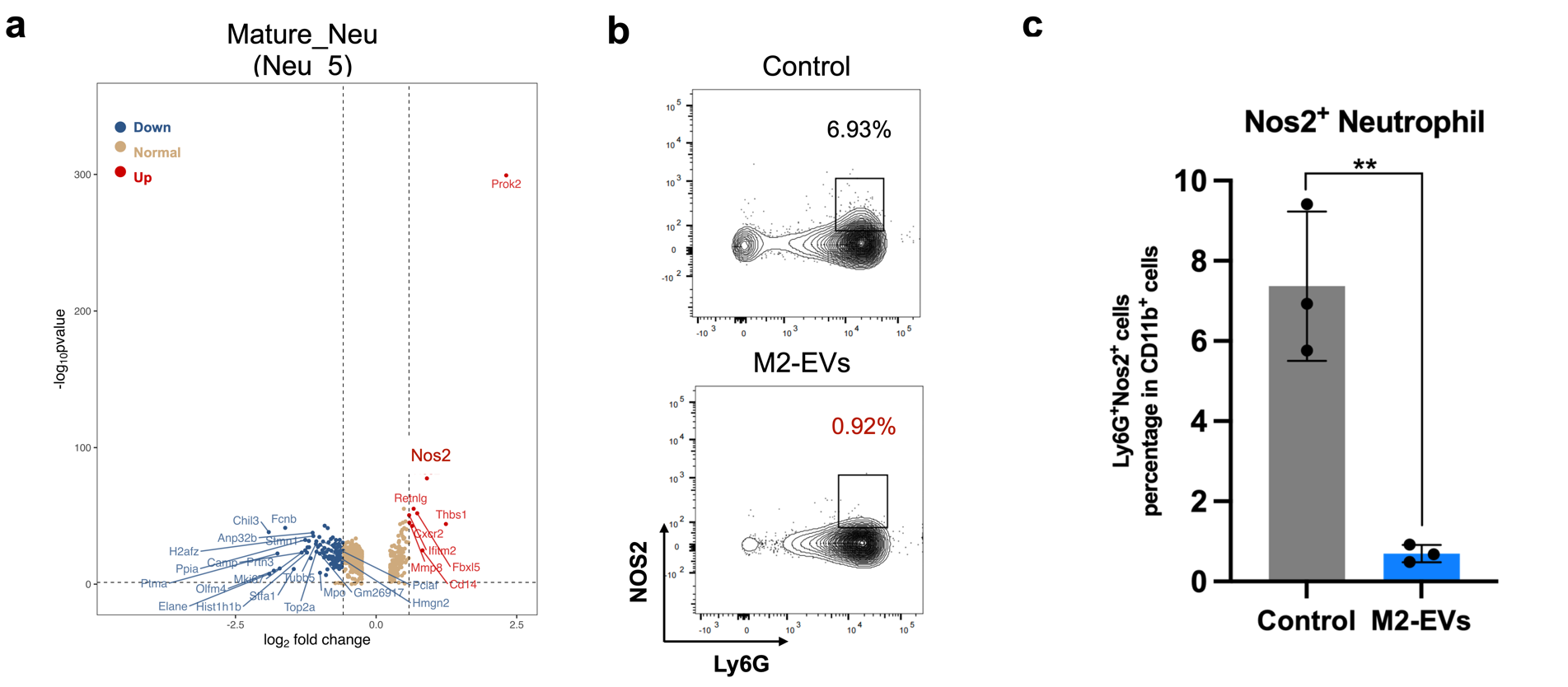
**

**Supplementary Figure 5.** **Identification of the changes in Nos2^+^ neutrophil subpopulations.** (a) Volcano plot displayed differential gene expression in Neu_5 compared to other neutrophil subpopulations. (b) Flow cytometry analysis on Ly6G^+^Nos2^+^ cells demonstrated the proportion changes of Nos2^+^ neutrophils in periodontal bone tissue. (c) Quantitative analysis of flow cytometry was performed to assess the proportion of Nos2^+^ neutrophils.


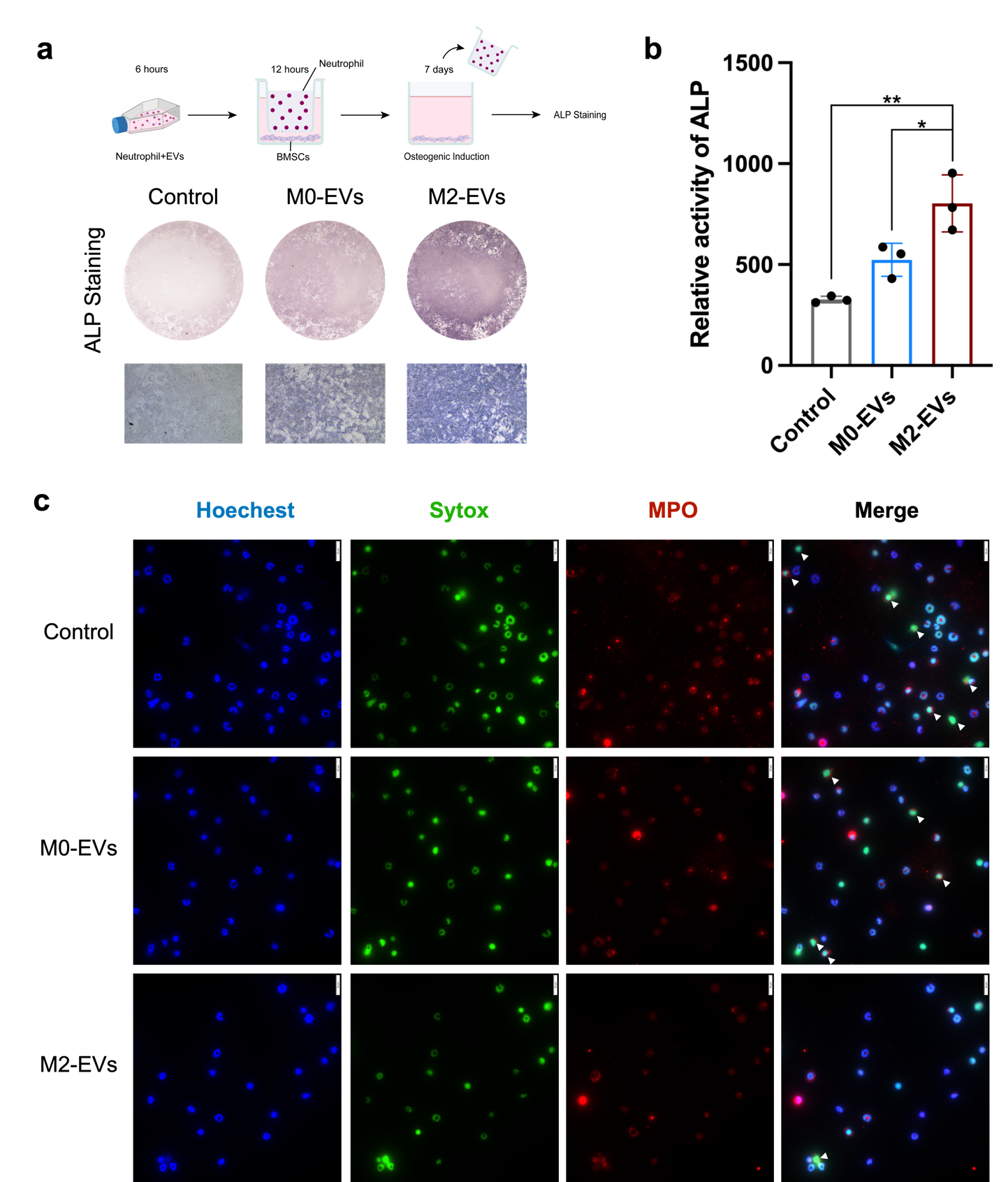


**Supplementary Figure 6. Pro-reparative role of immature neutrophils treated with M2-EVs.** (a) Alkaline phosphatase (ALP) staining to reveal the osteogenic potential of neutrophils pretreated with M2-EVs and co-cultured with bone marrow stem cells. (b) Quantitative analysis of ALP. (c) Representative confocal microscopy images showing neutrophil extracellular trap (NET) formation. Cells were stained for nuclei (Hoechest, blue), Sytox (green), and MPO (red). Scale bar indicates 20 μm. Error bars represent means ± SD from independent replicates (n＝3). ns, not significant, *p < 0.05; **p < 0.01. by Student’s t-test.


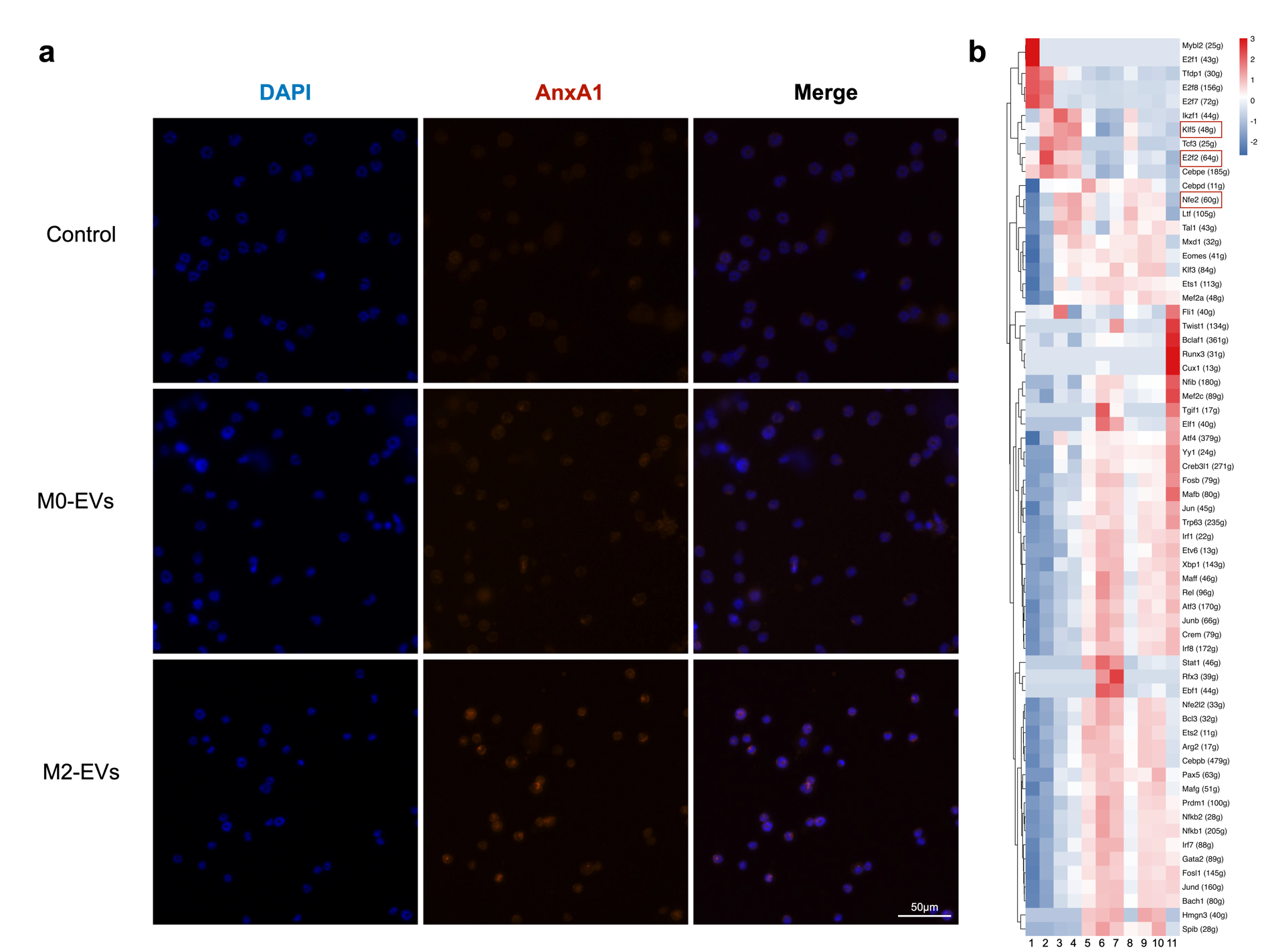
 **Supplementary Figure 7. Identification of the changes in Anxa1^hi^ neutrophil subpopulations.**  (a) Immunofluorescence revealing the proportion of Anxa1^hi^ neutrophils after M2-EVs treatment. (b) Transcriptional factors contributing to the neutrophil subpopulations.


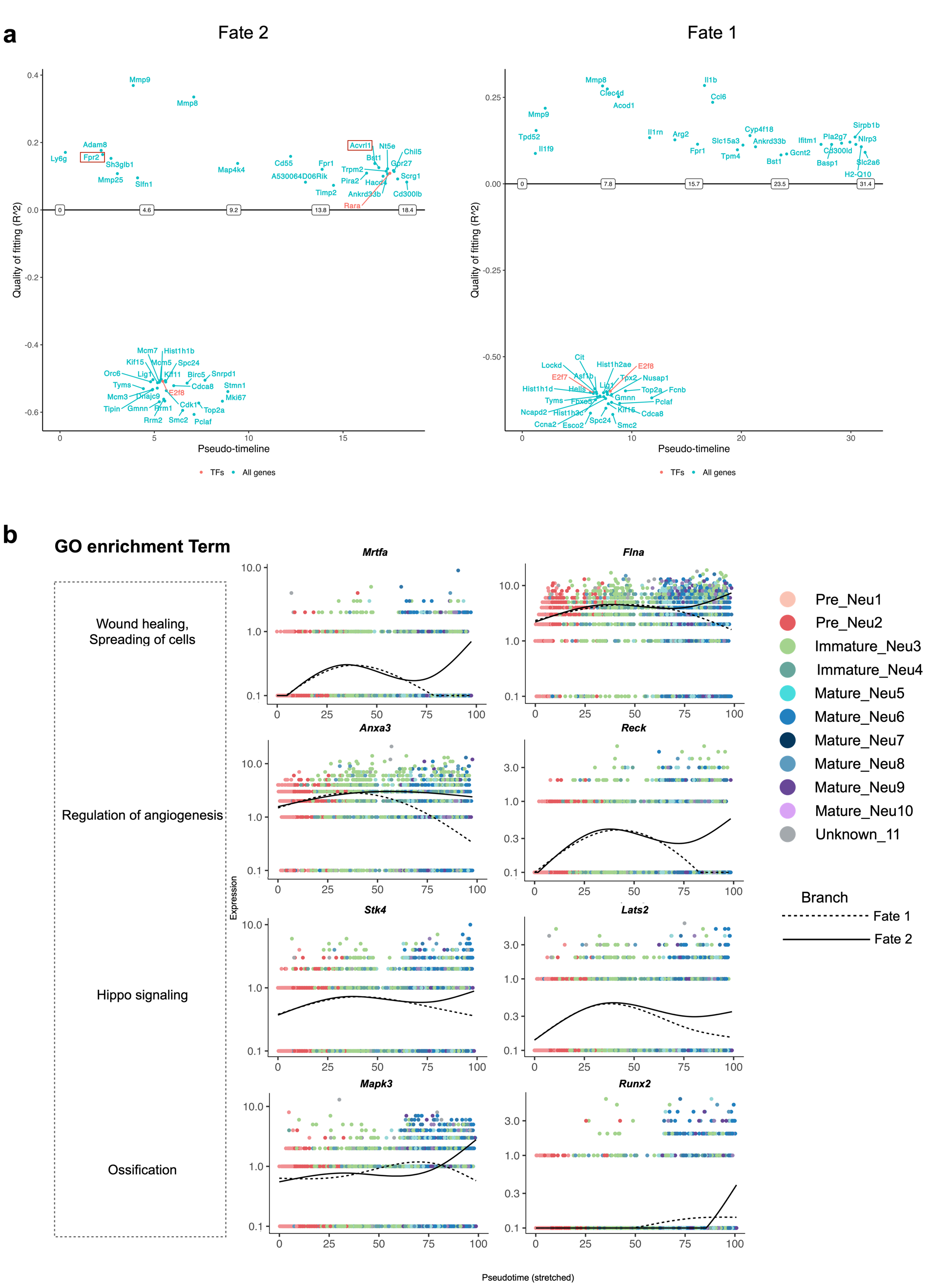


**Supplementary Figure 8.** **Gene expression dynamics during neutrophil differentiation and reparative transition.** (a) GeneSwitches analysis based on monocle trajectory illustrates the temporal changes in transcription factors and gene sets associated with Fate 1 and Fate 2. (b) Pseudotime-ordered single-cell expression trajectories illustrating gene expression dynamics along with the corresponding Gene Ontology (GO) enrichment terms in Fate 2.

**
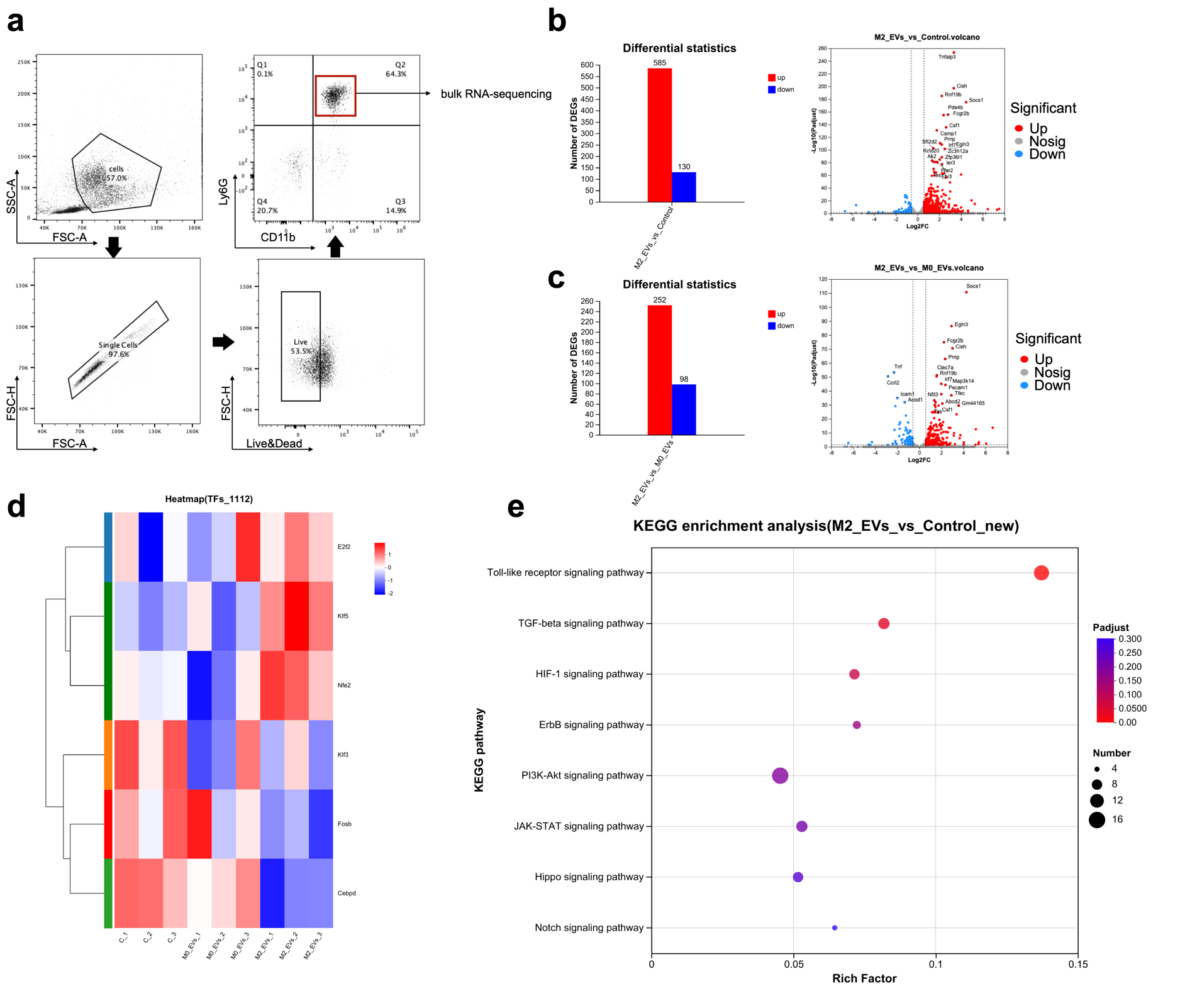
**

**Supplementary Figure 9. Bulk RNA-sequencing revealed the underlying mechanism of M2-EVs in modulating neutrophils.** (a) Gating strategy for Fluorescence-Activated Cell Sorting (FACS). (b, c) Differential expression analysis showing the number of differentially expressed genes (DEGs) and volcano plot illustrating gene expression changes (cutoff: p-value < 0.05, foldchange > 1.5 or < 1/1.5). (d) Representative transcription factors identified in the comparison between groups. (e) KEGG pathway analysis of differentially expressed genes between M2-EVs and control groups.


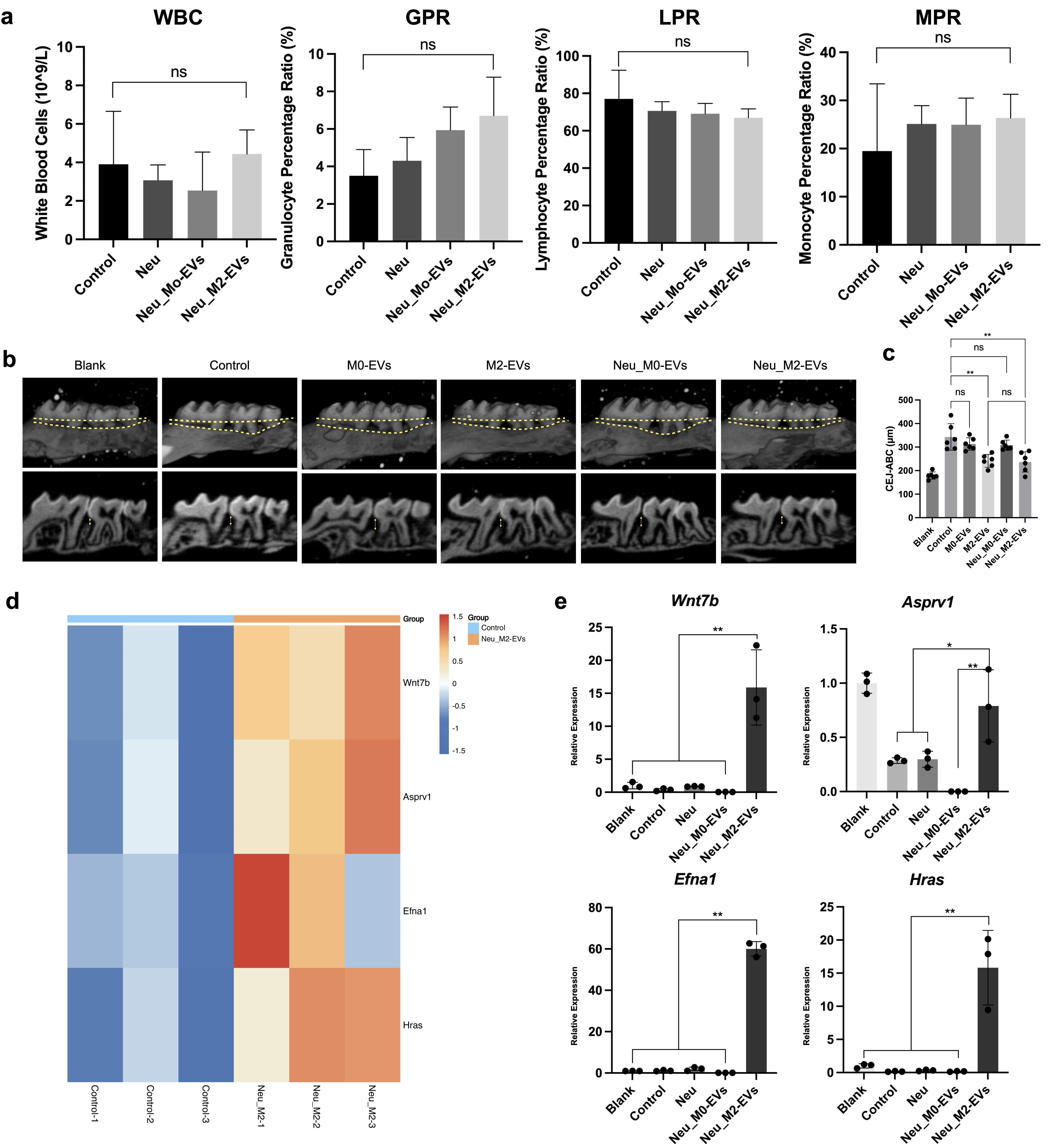


**Supplementary Figure 10. Evaluation of the effects following reprogrammed neutrophil therapy.** (a) Comprehensive analysis of peripheral blood parameters after neutrophil therapy. (b) Micro-CT scanning and three-dimensional reconstruction were performed to assess bone loss. (c) Bone loss was measured by the distance of CEJ-ABC in the second molar. (d) Heatmap of selected gene expression from GSEA pathway analysis. (e) qPCR analysis of representative gene expression including *Wnt7b, Asprv1, Efna1* and *Hras* between groups.

**
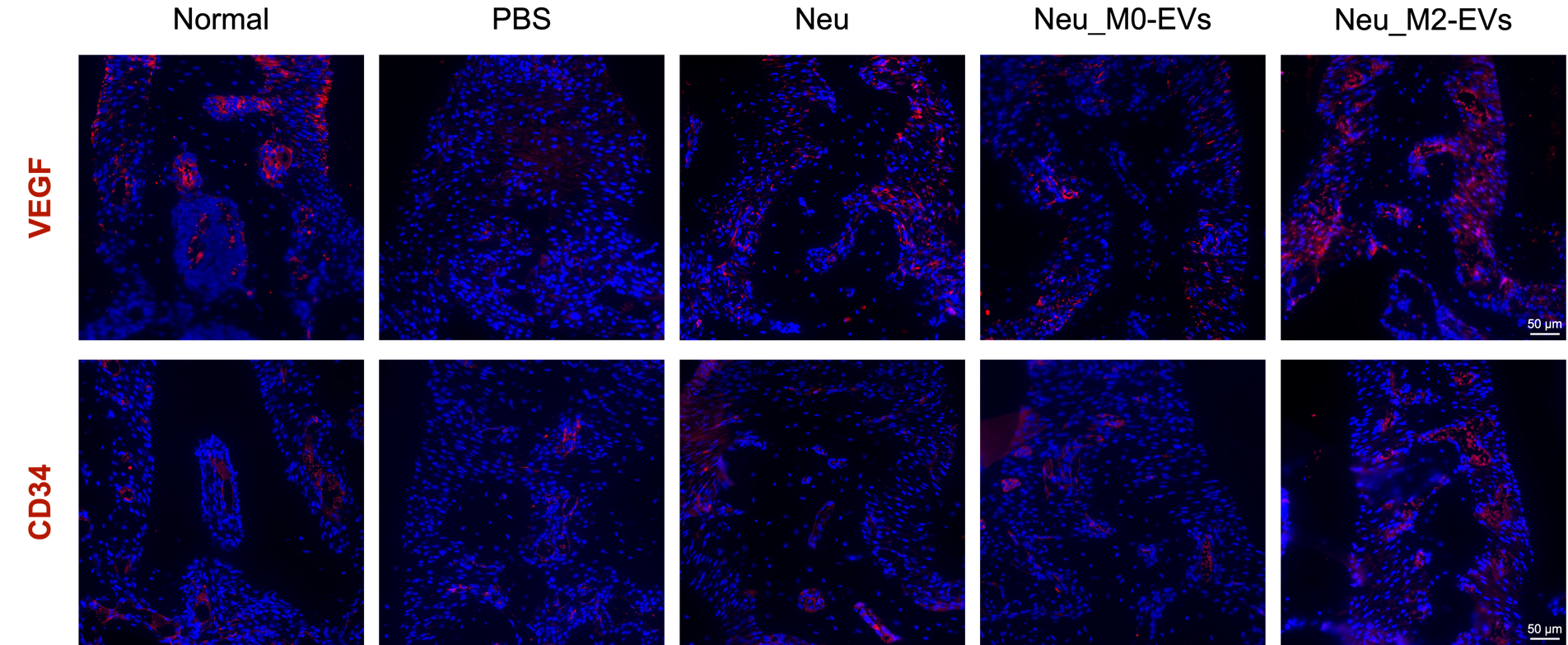
Supplementary Figure 11. Immunofluorescence staining of VEGF and CD34 showing angiogenesis signaling and endothelial progenitor cell distribution.**
